# Supplementary material for: Recording of patients’ mental health and quality of life-related outcomes in primary care: a cross-sectional study in the UK
Source: BMJ Open. 2022 Dec 21;12(12):e066949. doi: 10.1136/bmjopen-2022-066949 (PMC9772634; doi:10.1136/bmjopen-2022-066949)
Supplement: Author's manuscript [file bmjopen-2022-066949.draft_revisions.pdf]

BMJ Open is committed to open peer review. As part of this commitment we make the peer review history of every article we publish publicly available.

When an article is published we post the peer reviewers' comments and the authors' responses online. We also post the versions of the paper that were used during peer review. These are the versions that the peer review comments apply to.

The versions of the paper that follow are the versions that were submitted during the peer review process. They are not the versions of record or the final published versions. They should not be cited or distributed as the published version of this manuscript.

BMJ Open is an open access journal and the full, final, typeset and author-corrected version of record of the manuscript is available on our site with no access controls, subscription charges or pay-per-view fees (http://bmjopen.bmj.com).

If you have any questions on BMJ Open's open peer review process please email info.bmjopen@bmj.com

BMJ Open

Recording of patients' mental health and quality of life-related outcomes in primary care: a cross-sectional study in the UK

Journal:	*BMJ Open*
Manuscript ID	bmjopen-2022-066949
Article Type:	Original research
Date Submitted by the Author:	28-Jul-2022

Complete List of Authors:	Carreira, Helena; London School of Hygiene & Tropical Medicine, Department of Non-Communicable Disease Epidemiology<br>Dempsey, Harley; Medicines and Healthcare Products Regulatory Agency<br>Williams, Rachael; Clinical Practice Research Datalink<br>Bhaskaran, Krishnan; LSHTM, NCDE

Keywords:	PRIMARY CARE, MENTAL HEALTH, GENERAL MEDICINE (see Internal Medicine)

SCHOLARONE™
Manuscripts

I, the Submitting Author has the right to grant and does grant on behalf of all authors of the Work (as defined in the below author licence), an exclusive licence and/or a non-exclusive licence for contributions from authors who are: i) UK Crown employees; ii) where BMJ has agreed a CC-BY licence shall apply, and/or iii) in accordance with the terms applicable for US Federal Government officers or employees acting as part of their official duties; on a worldwide, perpetual, irrevocable, royalty-free basis to BMJ Publishing Group Ltd ("BMJ") its licensees and where the relevant Journal is co-owned by BMJ to the co-owners of the Journal, to publish the Work in this journal and any other BMJ products and to exploit all rights, as set out in our licence.

The Submitting Author accepts and understands that any supply made under these terms is made by BMJ to the Submitting Author unless you are acting as an employee on behalf of your employer or a postgraduate student of an affiliated institution which is paying any applicable article publishing charge ("APC") for Open Access articles. Where the Submitting Author wishes to make the Work available on an Open Access basis (and intends to pay the relevant APC), the terms of reuse of such Open Access shall be governed by a Creative Commons licence – details of these licences and which Creative Commons licence will apply to this Work are set out in our licence referred to above.

Other than as permitted in any relevant BMJ Author's Self Archiving Policies, I confirm this Work has not been accepted for publication elsewhere, is not being considered for publication elsewhere and does not duplicate material already published. I confirm all authors consent to publication of this Work and authorise the granting of this licence.

For peer review only

Recording of patients' mental health and quality of life-related outcomes in primary care: a cross-sectional study in the UK

Helena Carreira, MSc PhD, [1] Rachael Williams, MSc PhD,[2] Harley Dempsey, MSc,[3] Krishnan Bhaskaran, MSc PhD[4]

Authors' affiliations:

[1] Assistant Professor, Department of Non-Communicable Disease Epidemiology, Faculty of Epidemiology and Population Health, London School of Hygiene & Tropical Medicine, Keppel Street, London WC1E 7HT, United Kingdom. https://orcid.org/0000-0003-1538-2526

[2] Observational Research Team Leader, Clinical Practice Research Datalink (CPRD), Medicines and Healthcare products Regulatory Agency, 10th Floor, 10 South Colonnade, Canary Wharf, London E14 4PU, United Kingdom. https://orcid.org/0000-0001-9007-6292

[3] Clinical Study Manager, Clinical Practice Research Datalink (CPRD), Medicines and Healthcare products Regulatory Agency, 10th Floor, 10 South Colonnade, Canary Wharf, London E14 4PU, United Kingdom. https://orcid.org/0000-0001-9605-1264

[4] Professor, Department of Non-Communicable Disease Epidemiology, Faculty of Epidemiology and Population Health, London School of Hygiene & Tropical Medicine, Keppel Street, London WC1E 7HT, United Kingdom. https://orcid.org/0000-0001-5364-8757

Correspondence to:

Helena Carreira

Department of Non-communicable Disease Epidemiology

London School of Hygiene and Tropical Medicine

Keppel Street, London, WC1E 7HT, UK

Helena.carreira@lshtm.ac.uk

Keywords: anxiety, depression, quality of life, electronic health records, primary care, UK.

For peer review only - http://bmjopen.bmj.com/site/about/guidelines.xhtml

Abstract

Objective: To compare patient-reported anxiety, depression, and QoL outcomes, with data registered in patients' primary care electronic health record (EHR).

Design: Cross-sectional study.

Setting: Primary care.

Participants: 608 women registered in the Clinical Practice Research Datalink (CPRD) GOLD primary care database.

Primary and secondary outcome measures: Patient-reported data on anxiety, depression, and QoL, collected through postal questionnaires, and compared with coded information in EHR up to 2 years prior.

Results: Abnormal anxiety symptoms were reported by 118 of 599 who answered the relevant questions (21%); 59/118 (50%) had GP-recorded anxiolytic/antidepressant use, and 2 (1.6%) had anxiety coded in the EHR. 26/601 women (11%) reported depression symptoms, of whom 17 (65.4%) had GP-recorded antidepressant use and none had depression coded. 65 of 123 women reporting distress on the pain QoL domain (52.8%) had a corresponding record in the EHR <3 months before and 92 (74.8%) <24 months before. No patients reporting fatigue (n=157), sexual health problems (156), social avoidance (82) or cognitive problems (93) had corresponding codes in the EHR.

Conclusion: Many patients reporting mental health and QoL problems had no record of this in coded primary care data. This suggests that GPs may not always be aware of patient distress, implying missed opportunities for intervention, and that coded data does not fully reflect the burden of disease.

Keywords: mental health, quality of life, primary health care, United Kingdom

For peer review only - http://bmjopen.bmj.com/site/about/guidelines.xhtml

Strengths and Limitations

- A unique strength of this study is the ability to directly compare patient reported outcomes with data available in the electronic health records. Validated instruments were used to assess mental health outcomes. Outcomes in the primary care data were defined based on a systematic review of the codes.

- Limitations of this study include the lack of information for drugs sold over the counter, which are not captured by CPRD, and the use the possible use of anxiolytics and antidepressants for conditions other than anxiety/depression.

- Most of patient care is recorded using codes but GPs sometimes use other methods to keep records (e.g. free text entry) which are not available to us. Similarly, some codes in the patient records are unspecific (e.g., mood observations) and we could not assign a correspondence to domains of QoL.

For peer review only

Funding

H.C. was supported by an MRC Case Studentship in Epidemiology, jointly funded by UK Medical Research Council (MRC; https://mrc.ukri.org/) and the Clinical Practice Research Datalink (https://www.cprd.com/) at the Medicines and Healthcare Products Regulatory Agency (grant number MR/M016234/1 to H.C.). K.B. is funded by a Wellcome Senior Research Fellowship (grant number 220283/Z/20/Z) and previously held a Sir Henry Dale Fellowship jointly funded by Wellcome Trust (https://wellcome.ac.uk/) and the Royal Society (https://royalsociety.org/) (grant number 107737/Z/15/Z). The funders had no role in study design, data collection and analysis, decision to publish, or preparation of the manuscript.

Open Access

This research was funded in part by the Wellcome Trust [grant numbers 107737/Z/15/Z and 220283/Z/20/Z]. For the purpose of open access, the authors have applied a CC BY public copyright licence to any Author Accepted Manuscript version arising from this submission.

Conflict of interest

Dr Bhaskaran reports grants from Wellcome Trust, the Royal Society, Medical Research Council, and British Heart Foundation, outside the submitted work. Dr Williams and Ms Dempsey report that CPRD has financial relationships with its clients, including the London School of Hygiene and Tropical Medicine, in relation to providing access to research data and services outside the submitted work. HC has no conflict of interest to disclose.

For peer review only

Introduction

Quality of life (QoL) and mental health are amongst the most important outcomes for individuals, but the prevalence of problems is high [1, 2]. Improving QoL and reducing the mental health burden is challenging but there is consensus that public health strategies should include prevention, timely diagnosis, and optimising management and treatment of prevalent cases [3].

Early diagnosis and treatment of patients with adverse mental health outcomes is not always possible, in part because symptoms are often unspecific and go unrecognised, and because patients do not always seek care for mental health-related conditions [4, 5]. There has been a lack of research quantifying the burden of mental health and other QoL-related complaints that have not been picked up in primary care, and therefore may remain undiagnosed and untreated. One way of quantifying the gap between adverse mental health and QoL-related outcomes recorded in primary care, and those experienced by patients, is to directly collect patient-reported outcomes (PROs) and compare with information on the same outcomes in the clinical record. Under-recording of problems in primary care records could suggest lack of awareness by the general practitioner (GP) about the patient's lack of wellbeing, and thus a missed opportunity for care. Under-recording might also reflect inconsistent coding of mental health and QoL problems in the primary care record, with important implications for audit and research based on electronic health records (EHR).

In this study, we compared patient-reported information on symptoms of anxiety, depression, and QoL domains, with data for similar constructs registered in the patients' EHR. We used data from a previous study that collected PROs data from a convenience sample of women with and without history of breast cancer [6], and for whom EHR data were available.

For peer review only - http://bmjopen.bmj.com/site/about/guidelines.xhtml

Methods

Study design and sampling frame

We used a convenience sample of women with PRO data available from a previous study [6]. For the original study, primary care practices contributing with data to the Clinical Practice Research Datalink (CPRD) GOLD primary care database in August 2018 were invited to participate. CPRD GOLD includes EHR of patients attending general practices in the UK that use Vision software to manage patient's records. Data are entered in the patients' EHR by GPs during consultations using Read codes [7], which include information on symptoms, diagnoses, and prescriptions [8]. Patients registered with primary care practices that accepted to participate were considered potentially eligible for the study.

Patient eligibility criteria, selection and recruitment

A full description of eligibility and recruitment has been published elsewhere [6]. Briefly, inclusion criteria for the breast cancer survivors' group were 1) diagnosis of invasive breast cancer at least one year before (all stages) and 2) aged 18-80 years. To ensure that the recorded breast cancer was incident, we required one year of follow-up in CPRD prior to the breast cancer diagnosis. For the comparison group, inclusion criteria were 1) no history of cancer (except non-melanoma skin cancer), 2) aged 18-80 years, and 3) at least 2 years of follow-up data in CPRD (since we required one year of follow-up before and after cancer to be included in the breast cancer group). Exclusion criteria for both groups were 1) inability to complete a self-reported questionnaire (e.g. due to dementia) and 2) having had another (non-breast) cancer or having been treated for a non-invasive breast tumour.

The CPRD GOLD primary care database was used to identify all breast cancer survivors from participating practices, and a random sample of women with no prior cancer (frequency matched on age to breast cancer survivors) from the same practices. GPs applied inclusion and exclusion criteria (*vide* above), and sent the study materials to the eligible patients' addresses with a pre-paid envelope to return the questionnaires. Patients were recruited between January and November 2019.

Patient-reported outcomes

Anxiety and depression

For peer review only - http://bmjopen.bmj.com/site/about/guidelines.xhtml

Anxiety and depressive symptoms were measured with the Hospital Anxiety and Depression Scale (HADS) [9]. This is a 14-item self-reported screening tool for anxiety and depressive symptoms in the past week [9]. Based on their responses, we categorised patients as non-case (scores 0-7), borderline (scores 8-10) and probable case (scores 11-21) [9].

The QoL impact of anxiety and depression were measured with the respective domains in the Quality of Life in Adult Cancer Survivors Scale (QLACS) (see below).

Quality of life

QoL was assessed with QLACS [10]. This tool includes 47 items, divided in 7 generic domains (i.e. negative feelings; positive feelings; cognitive problems; pain; sexual function/interest; energy/fatigue; and social avoidance), and 5 cancer-specific domains (i.e. financial problems; benefits of cancer; distress-family; appearance; distress-recurrence) which are not considered further in this paper. Of the 7 generic QoL domains, 6 were considered suitable for comparison with data in the EHR because women with distress for these domains may visit their GP to seek help: 'negative feelings', 'cognitive problems', 'pain', 'sexual problems', 'fatigue' and 'social avoidance'. Each domain considered has 4 items on the QLACS questionnaire. Participants are instructed to answer in relation to the previous four weeks. Responses to each item are given on a Likert-type of scale that varies between 1 (never) and 7 (always); higher scores indicate poorer QoL.

To identify women who had high levels of distress for each domain, we calculated the mean response (i.e. the sum of the individual item scores divided by four; mean values range between one and seven). We considered a mean of ≥5 (corresponding to average replies of frequently, very often or always experiencing the stated symptom) to reflect distress in that domain. This was varied in sensitivity analyses (see below).

Outcomes recorded in electronic health records primary care data

We extracted the primary care EHR data for all participants. As PROs were collected between January and November 2019, we extracted data from the January 2020 version of CPRD GOLD, which included data from 1987 up to December 2019.

Anxiety and depression were defined using lists of Read codes from a systematic review [11]. For the QoL domains, we produced lists of Read codes closely related to the items in the QLACS domain (Supplementary Table 1). Read codes were used to identify women with these codes registered in their EHR in the 3, 6, 12 and 24 months prior to the date of last data collection from the practice. The last collection date varied from practice to practice, but was generally within three weeks of the database version (e.g. in the January 2020 version, the

For peer review only - http://bmjopen.bmj.com/site/about/guidelines.xhtml

date of last data collection from the practices was in median 20 days (inter-quartile range: 19-20) prior to 31 December 2019).

Data analysis

We calculated the proportion of women who reported high levels of distress in the questionnaires and had similar information in their EHR (i.e. sensitivity of the EHR for capturing patient-reported distress). To better understand the agreement between PROs and the EHR data, as a secondary analysis, we also calculated the proportion of women with codes indicating distress on each domain in their EHR that reported distress levels in the questionnaires (positive predictive value of the EHR for capturing patient-reported distress). Results were shown in tables and descriptively.

Sensitivity analysis

As we used an arbitrary cut-off to identify patients with poor QoL (mean domain-specific score of ≥5), two sensitivity analyses were conducted: 1) using a lower cut-off of ≥3; 2) considering a score of ≥5 on at least one item in the domain (rather than the mean) as reflecting distress. Finally, we explored whether breast cancer survivors had different results compared to women with no history of cancer.

Patient and Public Involvement

The authors are thankful to the cancer survivors involved with the Independent Cancer Patients' Voice (http://www.independentcancerpatientsvoice.org.uk/), a patient advocate group, for their comments on the study protocol.

For peer review only

Results

<u>*Characteristics of the participants*</u>

608 women from 40 primary care practices participated in the study (Table 1). Participants and non-participants were similarly distributed by country (England, Wales, Scotland, Northern Ireland) and deprivation. A quarter of the women had a higher education degree.

Table 1 Characteristics of the study participants. *

	All participants (N=608)	
	**N**	**%**
**Age at completion of questionnaire**		
34-59 years	174	28.6
60-69 years	210	34.5
≥70 years	224	36.8
**Highest education level**		
Up to GCSEs, O levels, or equivalent	205	33.7
A levels or equivalent	65	10.7
Trade or technical training	106	17.4
Undergraduate or post-graduate degree	160	26.3
Did not want to disclose	72	11.8
**Ethnicity**		
White	589	96.9
Asian / Asian British	7	1.2
Did not want to disclose	12	2.0
**IMD quintile**		
1 (least deprived)	124	20.4
2	90	14.8
3	81	13.3
4	239	39.3
5 (most deprived)	74	12.2
**Living arrangements**		
Not alone	458	75.3
Alone	138	22.7
Did not want to disclose	12	2.0
**Country**		
England	114	18.8
Northern Ireland	49	8.1
Scotland	188	30.9
Wales	257	42.3

IMD = Index of Multiple Deprivation

* Proportion may not add to 100% due to rounding

For peer review only

Anxiety and depression

Of the 599 women that replied to the anxiety subscale, 242 (40%) had borderline to abnormal symptoms (Table 2). Borderline to abnormal symptoms of depression were also reported by 92 (15%) of the 601 women that replied to the subscale for depression. Almost no women had Read codes for anxiety or depression registered in their EHR in the 24 months prior. However, 108/242 (45%) of those reporting anxiety symptoms were prescribed with an anxiolytic or antidepressant (for anxiety), and 51/92 (55%) of those reporting depression symptoms were prescribed an antidepressant.

In the QoL scale, 100 of the 608 (17%) women that replied to questionnaire had average replies of frequently, very often or always experiencing negative feelings (mean score ≥5). Only 1 patient (1%) had Read codes related to anxiety and/or depression recorded in their EHR in the 24 months prior, but 51 (51%) had an antidepressant and/or anxiolytic prescription.

Of the patients that had information about negative feelings in their EHR, only a minority reported distress in the questionnaires (Supplementary table 2).

Other QoL domains: Cognitive problems, fatigue, pain, sexual dysfunction, social avoidance

93/608 (16%) women reported high levels of distress related to cognitive problems, 156 (26%) to sexual dysfunction, 157 (26%) to fatigue/energy, and 82 (14%) to social avoidance (Table 3). No codes relevant to these domains were found in the patients' EHR up to 24 months prior. Using lower cut-offs to classify patients based in their QoL scores yielded similar results. Distress with pain was reported in the questionnaires by 123 (21%) of the women, and 65 (53%) of these had symptoms of pain and/or analgesic prescription recorded their EHR in the previous three months; this increased to 92 (75%) when a longer 24-month time window was used.

Sensitivity analyses

Results were similar to those of the main analysis when different criteria were used to define distress (see Table 2 and Table 3). There were no meaningful differences in the results between breast cancer survivors and women with no history of cancer (see Supplementary Table 3 and Supplementary Table 4).

For peer review only - http://bmjopen.bmj.com/site/about/guidelines.xhtml

Table 2 Capture of patient-reported anxiety and depression in patients' primary care records.

Patient reported outcomes			Read codes for symptoms/diagnoses in the patients' electronic health records by time prior to LCD								Relevant drug prescription by time prior to LCD *							
			3mo		6mo		12mo		24mo		3mo		6mo		12mo		24mo	
	N	%	N	%	N	%	N	%	N	%	N	%	N	%	N	%	N	%
**HADS**																		
**Anxiety**																		
Normal	357	59.6	0	0	0	0	0	0	0	0	53	14.9	55	15.4	67	18.8	84	23.5
Borderline	124	19.7	0	0	0	0	0	0	0	0	33	28.0	34	33.9	40	33.9	49	41.5
Abnormal	118	20.7	1	0.8	1	0.8	2	1.6	2	1.6	40	32.3	42	28.8	53	42.7	59	50.0
**Depression**																		
Normal	509	84.7	0		0		0		0		75	14.7	77	15.1	96	18.9	120	23.6
Borderline	66	4.3	0		0		0		0		26	39.4	26	39.4	30	45.5	34	51.5
Abnormal	26	11.0	0		0		0		0		13	50.0	13	50.0	16	61.5	17	65.4
**QLACS**																		
**Negative feelings**																		
≥5	100	16.8	1	1.0	1	1.0	1	1.0	1	1.0	36	36.0	37	37.0	47	47.0	51	51.0
≥3	386	64.7	1	0.3	1	0.3	2	0.5	2	0.5	89	23.1	90	23.3	109	28.2	129	33.4
1 item ≥5	227	37.3	1	0.4	1	0.4	1	0.4	1	0.4	65	28.6	66	29.1	82	36.1	95	41.9

PRO = Patient reported outcomes; HADS = Hospital Anxiety and Depression Scale; QLACS = Quality of Life in Adults Cancer Survivors Scale. LCD = last collection date for the practice; mo = months. * Anxiolytics or antidepressants for anxiety; antidepressants for depression.

For peer review only

For peer review only - http://bmjopen.bmj.com/site/about/guidelines.xhtml

Table 3 Capture of patient-reported QoL-related distress in patients' primary care records.

QoL Domain	Domain score	Patient reported outcomes		Relevant Read codes in the electronic health record*, by time prior to the date of last data collection							
				3mo		6mo		12mo		24mo	
		No. §	%	No.	%	No.	%	No.	%	No.	%
Cognitive problems	Average ≥5	93	15.5	0		0		0		0	
	Average ≥3	394	65.6	0		0		0		0	
	1 item ≥5	193	31.7	0		0		0		0	
Fatigue	Average ≥5	157	26.1	0		0		0		0	
	Average ≥3	472	78.5	0		0		0		0	
	1 item ≥5	536	88.2	0		0		0		0	
Physical pain	Average ≥5	123	20.6	65	52.8	70	56.9	82	66.7	92	74.8
	Average ≥3	330	55.4	106	32.1	116	35.2	152	46.1	186	56.4
	1 item ≥5	231	38.0	86	37.2	94	40.7	120	52.0	142	61.5
Sexual dysfunction	Average ≥5	156	25.7	0		0		0		0	
	Average ≥3	377	62.0	0		0		0		0	
	1 item ≥5	304	50.0	0		0		0		0	
Social avoidance	Average ≥5	82	13.5	0		0		0		0	
	Average ≥3	294	48.4	0		0		0		0	
	1 item ≥5	196	32.2	0		0		0		0	

QoL = quality of life; mo = months. * Severe cognitive dysfunction was an exclusion criterion for the study. § 608 women participated in the study; due to missing data for some items, the number of women included in the denominator varies slightly by domain. *Relevant Read codes were codes for cognitive impairment, dementia and dementia specific drugs (cognitive problems domain); low energy, tiredness (fatigue domain); pain, pain syndromes, analgesics prescriptions (pain domain); low libido, anorgasm, vaginismus (sexual dysfunction); social isolation and avoidance (social avoidance domain).

For peer review only

Discussion

Summary

Most patients who reported clinically relevant symptoms of anxiety and depression, and distress with cognitive problems, fatigue, physical pain, sexual dysfunction and social avoidance, did not have clinical codes for these conditions in their primary care EHR. This suggests that in some cases GPs may be unaware of problems adversely affecting their patients' QoL. Our results may also be partly explained by inconsistent coding, as evident from the number of women in receipt of medications for anxiety and depression, despite no diagnostic codes being present in the EHR. In these cases, GPs were evidently aware of the patient's condition but had not entered a diagnostic code into the EHR, which could lead to misleading information when routine coded data are used for audit and research.

Strengths and limitations

The ability to compare PROs with data available in the EHR represents a unique strength of this study. To our knowledge, no previous study has reported on this comparison. However, this study had limitations. The date of questionnaire completion was not collected, and we could identify, precisely, the consultations that corresponded to when the PROs were evaluated. This probably had little impact in the results, since the results of the analyses going back 24 months were not distinctively different from the results for 3 and 6 months. Our approach to identify patients experiencing distress on specific QLACS domains used score thresholds that were not validated. However, sensitivity analysis using different cut-offs showed generally the same patterns. CPRD only captures drugs prescribed to patients, and widely used drugs for pain and fatigue are sold over-the-counter. We assumed that anxiolytics and antidepressants were taken for anxiety/depression, but we cannot rule out that some were for other indications such as pain or insomnia. The comparison for cognitive problems was limited by the need to exclude patients unable to reply to a self-reported questionnaire, and we cannot rule out that GPs may have been overly strict in applying this exclusion criterion, excluding mild cognitive impairment. Our results are based on a convenience sample of breast cancer and non-cancer controls and may not be generalisable to the general population; however, results were similar in our sensitivity analysis comparing results between the two groups. We compared PROs with information coded in the EHR; while most of patient care is coded using records, GPs sometimes use other methods to keep records (e.g. free text entry) which are not available to us. Similarly, some codes in the EHR are unspecific (e.g. mood observations) and we could not assign a correspondence to domains of QoL. It is possible that we underestimated, in some cases, the awareness of the GP about the patients' wellbeing.

For peer review only - http://bmjopen.bmj.com/site/about/guidelines.xhtml

Comparison with existing literature

Only one in 3 patients that reported distressing levels of negative feelings had similar information recorded in their EHR in the previous 3 months. This is consistent with patients often not seeking primary care for anxiety and depression [12]. Approximately one-half of the women that reported poor QoL related to pain had related information in the EHR in the previous 3 months. This may be partly explained by patients self-treating pain with widely used over-the-counter treatments such as paracetamol and ibuprofen. Conversely, the higher recording of pain compared to negative feelings could be related to patients more often seeking help for concerns perceived as being amenable to treatment.

We did not find any records of cognitive dysfunction, social avoidance, sexual dysfunction or fatigue in the EHR of the participating women in the previous 24 months. An absence of entries for social avoidance is plausible; Read codes for social avoidance have seldom been used in the database. A lack of records for sexual dysfunction is in keeping with evidence that only a small proportion of people contact GPs for issues related to sexual function [13]. The lack of coded records for cognitive dysfunction and fatigue was more unexpected. It is possible that GPs systematically excluded people with mild cognitive dysfunction. For fatigue, a manual review of all entries in the EHR of patients that reported distressing levels of fatigue revealed a pattern of multimorbidity, almost always with diagnoses where fatigue is implicit (e.g., heart failure, chronic obstructive pulmonary disease), but no explicit codes for fatigue.

Implications for research and/or practice

It is important to raise awareness that patients may not always actively report their distress. Even when GPs are aware of health issues, they are not always coded in the patient record, and thus EHRs have low sensitivity to detect patients experiencing poor QoL at a particular point in time. Studies investigating anxiety and depression should consider prescriptions as well as clinical codes, as many patients were prescribed anxiolytics and antidepressants without having a Read code for these conditions.

Conclusion

We found substantial under-recording of mental-health and QoL-related distress in coded primary care data, suggesting that there may be missed opportunities to provide support to patients in need. In addition, there may be inconsistent coding of known conditions, meaning that studies of mental-health and QoL-related outcomes using EHR databases likely underestimate the absolute burden of these outcomes in the population.

For peer review only - http://bmjopen.bmj.com/site/about/guidelines.xhtml

Ethics approval and consent to participate

This study was approved by the East of England - Cambridge South Research Ethics Committee (Ref: 17/EE/0403), the London School of Hygiene and Tropical Medicine Interventions Research Ethics Committee (Ref: 14417) and the Health Research Authority and Health and Care Research Wales (IRAS Project ID: 224561). Implicit patient consent was obtained when the patient posted the completed questionnaires.

Data sharing statement

This study is based in part on data from the Clinical Practice Research Datalink obtained under licence from the UK Medicines and Healthcare products Regulatory Agency. The terms of our licence to access the data preclude us from sharing individual patient data with third parties. The electronic health records raw data may be requested directly from CPRD following their usual procedures.

Contributorship Statement

HC, RW, and KB designed the study. HC, RW, and KB obtained ethical approvals. HD, HC, RW, and KB managed data collection. HC entered the data and performed analyses. All authors revised the manuscript for important intellectual content and approved the final version of the manuscript.

For peer review only

References

1. *Quality of Life - The assessment, analysis and reporting of patient-reported outcomes*. third ed. 2016, UK: John Wiley & Sons, Ltd.

2. Institute for Health Metrics and Evaluation. *GBD Compare: Years Lived with Disability, United Kingdom, 2019*. [cited 2021; Available from: https://vizhub.healthdata.org/gbd-compare/.

3. Patel V, S Saxena, C Lund, G Thornicroft, et al. The Lancet Commission on global mental health and sustainable development. Lancet 2018:392:1553-1598.

4. Campion J, A Javed, C Lund, N Sartorius, et al. Public mental health: required actions to address implementation failure in the context of COVID-19. Lancet Psychiatry 2022:9:169-182.

5. Alonso J, Z Liu, S Evans-Lacko, E Sadikova, et al. Treatment gap for anxiety disorders is global: Results of the World Mental Health Surveys in 21 countries. Depress Anxiety 2018:35:195-208.

6. Carreira H, R Williams, H Dempsey, S Stanway, et al. Quality of life and mental health in breast cancer survivors compared with non-cancer controls: a study of patient-reported outcomes in the United Kingdom. J Cancer Surviv 2021:15:564-575.

7. Chisholm J. The Read clinical classification. BMJ 1990:300:1092.

8. Herrett E, AM Gallagher, K Bhaskaran, H Forbes, et al. Data Resource Profile: Clinical Practice Research Datalink (CPRD). Int J Epidemiol 2015:44:827-36.

9. Zigmond AS and RP Snaith. The hospital anxiety and depression scale. Acta Psychiatr Scand 1983:67:361-70.

10. Avis NE, KW Smith, S McGraw, RG Smith, et al. Assessing quality of life in adult cancer survivors (QLACS). Qual Life Res 2005:14:1007-23.

11. Carreira H, R Williams, H Strongman, and K Bhaskaran. Identification of mental health and quality of life outcomes in primary care databases in the UK: a systematic review. BMJ Open 2019:9:e029227.

12. Clement S, O Schauman, T Graham, F Maggioni, et al. What is the impact of mental health-related stigma on help-seeking? A systematic review of quantitative and qualitative studies. Psychol Med 2015:45:11-27.

13. Mercer CH, KA Fenton, AM Johnson, K Wellings, et al. Sexual function problems and help seeking behaviour in Britain: national probability sample survey. BMJ 2003:327:426-7.

For peer review only - http://bmjopen.bmj.com/site/about/guidelines.xhtml

Supplementary Table 1 Matching between QoL domain and information in the EHRs.

QoL domain	QLACS Items	Search in the EHR for Read codes* related to:
Negative feelings	19 Bothered by mood swings<br>7 Felt blue or depressed<br>9 Worried about little things<br>24 Felt anxious	Depression and/or anxiety (disorders and symptoms), antidepressants, or anxiolytic prescription
Cognitive problems	3 Bothered by having a short attention span<br>4 Had trouble remembering things<br>2 Difficulty doing things requiring concentration<br>23 Bothered by forgetting what started to do	Cognitive impairment; cognitive dysfunction symptoms; dementia*; dementia-specific drug*.
Physical pain	13 Bothered by pain preventing activities<br>17 Mood disrupted by pain or its treatment<br>27 Pain interfered with social activities<br>21 Had aches or pains	Pain; painful conditions; prescriptions of analgesics.
Sexual problems	16 Lacked interest in sex<br>26 Avoided sexual activity<br>12 Dissatisfied with sex life<br>10 Bothered by inability to function sexually	Low libido; anorgasmia; vaginismus.
Fatigue	11 Lacked energy to do things wanted to<br>14 Felt tired a lot<br>1 Had energy to do things wanted to do<br>5 Felt fatigued	Low energy; tiredness.
Social avoidance	18 Avoided social gatherings<br>20 Avoided friends<br>25 Reluctant to meet new people<br>15 Reluctant to start new relationships	Social isolation, or social avoidance.

* Definitions were based on a comprehensive systematic review of the studies that defined mental health and quality of life-related outcomes in primary care databases of electronic health records [11].

QLACS = Quality of Life in Adult Cancer Survivors Scale; EHR = electronic health records.

For peer review only

Supplementary Table 2 Patient reported outcomes of women with codes related to distress in their primary care record.

			**Patients with info for the domain in EHR**		**Patients scoring as distressed, according to patient-reported data**					
					≥5		≥3		At least one item 5	
**Domain**	**Read codes related to:**	**Time prior to LDC**	**No. §**	**%**	**No.**	**%**	**No.**	**%**	**No.**	**%**
Negative feelings	Depression and/or anxiety (disorders and symptoms), antidepressants, or anxiolytic prescription.	3 mo.	118	19.4	36	31.3	89	77.4	65	55.1
		6 mo.	120	19.7	37	31.6	90	76.9	66	55.0
		12 mo.	146	24.0	47	33.1	109	76.8	82	56.2
		24 mo.	170	28.5	51	30.0	129	75.9	95	54.3
Cognitive problems	Cognitive impairment; cognitive dysfunction symptoms; dementia; dementia-specific drug*	3 mo.	0		-		-		-	
		6 mo.	0		-		-		-	
		12 mo.	0		-		-		-	
		24 mo.	0		-		-		-	
Fatigue	Low energy; tiredness.	3 mo.	0		-		-		-	
		6 mo.	0		-		-		-	
		12 mo.	0		-		-		-	
		24 mo.	0		-		-		-	
Physical pain	Pain; painful conditions; prescriptions of analgesics.	3 mo.	138	37.2	65	48.2	106	78.5	86	62.3
		6 mo.	150	24.7	70	47.6	116	78.9	94	62.7
		12 mo.	203	34.1	82	40.4	152	74.9	120	58.0
		24 mo.	264	43.4	92	35.5	186	71.8	142	53.8
Sexual dysfunction	Low libido; anorgasm; vaginismus.	3 mo.	0		-		-		-	
		6 mo.	0		-		-		-	
		12 mo.	0		-		-		-	
		24 mo.	0		-		-		-	
Social avoidance	Social isolation; social avoidance.	3 mo.	0		-		-		-	
		6 mo.	0		-		-		-	
		12 mo.	0		-		-		-	
		24 mo.	0		-		-		-	

EHR = electronic health records; QoL = quality of life; mo = months. * Severe cognitive dysfunction was an exclusion criterion for the study. § 608 women participated in the study; due to missing data for some items, the number of women included in the denominator varies slightly by domain.

For peer review only

Supplementary Table 3 Patient-reported outcomes from women with history of breast cancer and non-cancer controls.

			PROs				Patients scoring above a given threshold in the PRO study that had domain-related information in EHRs, by time prior to the last data collection for the practice															
		Mean domain cut-off	Breast cancer survivors		Non-cancer controls		Breast cancer survivors								Non-cancer controls							
							3mo		6mo		12mo		24mo		3mo		6mo		12mo		24mo	
**QLACS Domain**	**Read codes for**		**No. §**	**%**	**No. §**	**%**	**No.**	**%**	**No.**	**%**	**No.**	**%**	**No.**	**%**	**No.**	**%**	**No.**	**%**	**No.**	**%**	**No.**	**%**
Negative feelings	Depression and/or anxiety (inc. symptoms), antidepressants, or anxiolytic prescription	≥5	62	17.4	38	15.1	23	37.1	23	37.1	29	46.8	32	51.6	13	34.2	14	36.8	18	47.4	19	50.0
		≥3	231	64.9	155	61.5	58	25.1	58	25.1	70	30.3	82	35.5	31	20.0	32	20.7	39	25.2	47	30.3
		1 item ≥5	140	39.3	87	34.5	42	30.0	42	30.0	53	37.9	61	43.6	23	26.4	24	27.6	29	33.3	34	39.1
Cognitive problems	Cognitive dysfunction; dementia; dementia-specific drug.*	≥5	61	17.1	32	12.7	0		0		0		0		0		0		0		0	
		≥3	238	66.9	156	61.9	0		0		0		0		0		0		0		0	
		1 item ≥5	123	34.6	70	27.8	0		0		0		0		0		0		0		0	
Physical pain	Pain; painful conditions; prescriptions of analgesics.	≥5	72	20.2	51	20.2	65	52.9	70	56.9	82	66.7	92	74.8	28	54.9	30	58.8	37	72.6	40	78.4
		≥3	194	54.5	136	54.0	106	32.1	116	35.2	152	46.1	186	56.4	42	30.9	45	33.1	65	47.8	77	56.6
		1 item ≥5	144	40.5	87	34.5	86	37.2	94	40.7	120	52.0	142	61.5	33	37.9	36	41.4	47	54.0	52	59.8
Sexual dysfunction	Low libido; anorgasm; vaginismus.	≥5	107	30.1	49	19.4	0		0		0		0		0		0		0		0	
		≥3	234	65.7	143	56.8	0		0		0		0		0		0		0		0	
		1 item ≥5	196	55.1	108	42.9	0		0		0		0		0		0		0		0	
Fatigue	Low energy; tiredness.	≥5	104	29.2	53	21.0	0		0		0		0		0		0		0		0	
		≥3	283	79.5	189	75.0	0		0		0		0		0		0		0		0	
		1 item ≥5	318	89.3	218	86.5	0		0		0		0		0		0		0		0	
Social avoidance	Social isolation; social avoidance.	≥5	50	14.0	32	12.7	0		0		0		0		0		0		0		0	
		≥3	171	48.0	123	48.8	0		0		0		0		0		0		0		0	
		1 item ≥5	125	35.1	71	28.2	0		0		0		0		0		0		0		0	

EHR = electronic health records; QoL = Health-Related Quality of Life; mo. = month. * Severe cognitive dysfunction was an exclusion criterion for the study. § 608 women participated in the study; due to missing data for some items, the number of women included in the denominator varies slightly by domain.

For peer review only

Supplementary Table 4 Patient-reported outcomes of patients with codes related to distress in their primary care record, by history of breast cancer.

			Patients with info for the domain in EHR				Patients scoring as distressed, according to patient-reported data												
							**Breast cancer survivors**						**Non-cancer controls**					
			**Breast cancer survivors**		**Non-cancer controls**		≥5		≥3		At least one item 5		≥5		≥3		At least one item 5	
**Domain**	**Read codes related to:**	**Time prior to LDC**	**No. §**	**%**	**No. §**	**%**	**No.**	**%**	**No.**	**%**	**No.**	**%**	**No.**	**%**	**No.**	**%**	**No.**	**%**
Negative feelings	Depression and/or anxiety (disorders and symptoms), antidepressants, or anxiolytics.	3 mo.	75	21.7	40	15.9	23	30.7	58	77.3	42	54.6	13	32.5	31	77.5	23	56.1
		6 mo.	76	22.0	41	16.3	23	30.3	58	76.3	42	53.9	14	34.2	32	78.1	24	57.1
		12 mo.	92	26.6	50	19.9	29	31.5	70	76.1	53	55.8	18	36.0	39	78.0	29	33.3
		24 mo.	109	31.5	61	24.3	32	29.4	82	75.2	61	54.0	19	31.2	47	77.1	34	54.8
Cognitive problems	Cognitive impairment; dementia; specific drugs*	3 mo.	0				-		-		-		-		-		-	
		6 mo.	0				-		-		-		-		-		-	
		12 mo.	0				-		-		-		-		-		-	
		24 mo.	0				-		-		-		-		-		-	
Physical pain	Pain; painful conditions; prescriptions of analgesics.	3 mo.	89	25.0	49	19.4	37	41.6	64	71.9	53	59.6	28	57.1	42	85.7	33	67.4
		6 mo.	98	27.5	52	20.6	40	40.8	71	72.5	58	59.2	30	57.7	45	86.5	36	69.2
		12 mo.	127	35.7	80	31.8	45	35.4	87	68.5	73	50.7	37	46.3	65	81.3	47	58.8
		24 mo.	162	45.5	102	40.5	52	32.1	109	67.3	90	55.6	40	39.2	77	75.5	52	51.0
Sexual dysfunction	Low libido; anorgasm; vaginismus.	3 mo.	0				-		-		-		-		-		-	
		6 mo.	0				-		-		-		-		-		-	
		12 mo.	0				-		-		-		-		-		-	
		24 mo.	0				-		-		-		-		-		-	
Fatigue	Low energy; tiredness.	3 mo.	0				-		-		-		-		-		-	
		6 mo.	0				-		-		-		-		-		-	
		12 mo.	0				-		-		-		-		-		-	
		24 mo.	0				-		-		-		-		-		-	
Social avoidance	Social isolation; social avoidance.	3 mo.	0				-		-		-		-		-		-	
		6 mo.	0				-		-		-		-		-		-	
		12 mo.	0				-		-		-		-		-		-	
		24 mo.	0				-		-		-		-		-		-	

EHR = electronic health records; QoL = Health-Related Quality of Life; mo. = month. * Severe cognitive dysfunction was an exclusion criterion for the study. § 608 women participated in the study; due to missing data for some items, the number of women included in the denominator varies slightly by domain.

For peer review only

BMJ Open

Recording of patients' mental health and quality of life-related outcomes in primary care: a cross-sectional study in the UK

Journal:	*BMJ Open*
Manuscript ID	bmjopen-2022-066949.R1
Article Type:	Original research
Date Submitted by the Author:	20-Oct-2022
Complete List of Authors:	Carreira, Helena; London School of Hygiene & Tropical Medicine, Department of Non-Communicable Disease Epidemiology<br>Williams, Rachael; Clinical Practice Research Datalink<br>Dempsey, Harley; Medicines and Healthcare Products Regulatory Agency<br>Bhaskaran, Krishnan; LSHTM, NCDE
**Primary Subject Heading**:	General practice / Family practice
Secondary Subject Heading:	Epidemiology
Keywords:	PRIMARY CARE, MENTAL HEALTH, GENERAL MEDICINE (see Internal Medicine)

SCHOLARONE™
Manuscripts

I, the Submitting Author has the right to grant and does grant on behalf of all authors of the Work (as defined in the below author licence), an exclusive licence and/or a non-exclusive licence for contributions from authors who are: i) UK Crown employees; ii) where BMJ has agreed a CC-BY licence shall apply, and/or iii) in accordance with the terms applicable for US Federal Government officers or employees acting as part of their official duties; on a worldwide, perpetual, irrevocable, royalty-free basis to BMJ Publishing Group Ltd ("BMJ") its licensees and where the relevant Journal is co-owned by BMJ to the co-owners of the Journal, to publish the Work in this journal and any other BMJ products and to exploit all rights, as set out in our licence.

The Submitting Author accepts and understands that any supply made under these terms is made by BMJ to the Submitting Author unless you are acting as an employee on behalf of your employer or a postgraduate student of an affiliated institution which is paying any applicable article publishing charge ("APC") for Open Access articles. Where the Submitting Author wishes to make the Work available on an Open Access basis (and intends to pay the relevant APC), the terms of reuse of such Open Access shall be governed by a Creative Commons licence – details of these licences and which Creative Commons licence will apply to this Work are set out in our licence referred to above.

Other than as permitted in any relevant BMJ Author's Self Archiving Policies, I confirm this Work has not been accepted for publication elsewhere, is not being considered for publication elsewhere and does not duplicate material already published. I confirm all authors consent to publication of this Work and authorise the granting of this licence.

For peer review only

Recording of patients' mental health and quality of life-related outcomes in primary care: a cross-sectional study in the UK

Helena Carreira, MSc PhD, [1] Rachael Williams, MSc PhD,[2] Harley Dempsey, MSc,[3] Krishnan Bhaskaran, MSc PhD[4]

Authors' affiliations:

[1] Assistant Professor, Department of Non-Communicable Disease Epidemiology, Faculty of Epidemiology and Population Health, London School of Hygiene & Tropical Medicine, Keppel Street, London WC1E 7HT, United Kingdom. https://orcid.org/0000-0003-1538-2526

[2] Observational Research Team Leader, Clinical Practice Research Datalink (CPRD), Medicines and Healthcare products Regulatory Agency, 10th Floor, 10 South Colonnade, Canary Wharf, London E14 4PU, United Kingdom. https://orcid.org/0000-0001-9007-6292

[3] Clinical Study Manager, Clinical Practice Research Datalink (CPRD), Medicines and Healthcare products Regulatory Agency, 10th Floor, 10 South Colonnade, Canary Wharf, London E14 4PU, United Kingdom. https://orcid.org/0000-0001-9605-1264

[4] Professor, Department of Non-Communicable Disease Epidemiology, Faculty of Epidemiology and Population Health, London School of Hygiene & Tropical Medicine, Keppel Street, London WC1E 7HT, United Kingdom. https://orcid.org/0000-0001-5364-8757

Correspondence to:

Helena Carreira

Department of Non-communicable Disease Epidemiology

London School of Hygiene and Tropical Medicine

Keppel Street, London, WC1E 7HT, UK

Helena.carreira@lshtm.ac.uk

Keywords: anxiety, depression, quality of life, electronic health records, primary care, UK.

For peer review only

Abstract

Objective: To compare patient-reported anxiety, depression, and QoL outcomes, with data registered in patients' primary care electronic health record (EHR).

Design: Cross-sectional study.

Setting: Primary care.

Participants: 608 women registered in the Clinical Practice Research Datalink (CPRD) GOLD primary care database (a convenience sample using data from a previous study on 356 breast cancer survivors (8.1 years post diagnosis) and 252 women with no prior cancer).

Primary and secondary outcome measures: Patient-reported data on anxiety, depression, and QoL, collected through postal questionnaires, and compared with coded information in EHR up to 2 years prior.

Results: Abnormal anxiety symptoms were reported by 118 of 599 who answered the relevant questions (21%); 59/118 (50%) had GP-recorded anxiolytic/antidepressant use, and 2 (1.6%) had anxiety coded in the EHR. 26/601 women (11%) reported depression symptoms, of whom 17 (65.4%) had GP-recorded antidepressant use and none had depression coded. 65 of 123 women reporting distress on the pain QoL domain (52.8%) had a corresponding record in the EHR <3 months before and 92 (74.8%) <24 months before. No patients reporting fatigue (n=157), sexual health problems (156), social avoidance (82) or cognitive problems (93) had corresponding codes in the EHR. There were no meaningful differences between breast cancer survivors and women with no prior history of cancer.

Conclusion: Many patients reporting mental health and QoL problems had no record of this in coded primary care data. This suggests that coded data does not fully reflect the burden of disease. Further research is needed to understand whether or not GPs are aware of patient distress in cases where codes have not been recorded.

Keywords: mental health, quality of life, primary health care, United Kingdom

For peer review only

Strengths and limitations of this study

- A strength of this study comes from the use of the Clinical Practice Research Datalink GOLD primary care database to select participants for the study, as it enabled the comparison of patient-reported outcomes with the data that had been routinely recorded in their electronic health record.
- Patient-reported outcomes were assessed using validated tools and identification of data in the coded electronic health records was based in a systematic review of Read codes.
- Limitations of this study include the lack of information for drugs sold over the counter, which are not captured by CPRD, and that we could not distinguish when anxiolytics and antidepressants may have been used for conditions other than anxiety/depression.
- Most patient care is recorded using codes but General Practitioners sometimes use other methods to keep records (e.g., free text entry) which are not available to us. Similarly, some codes in the patient records are unspecific (e.g., mood observations) and we could not assign a correspondence to domains of QoL.
- This study included only adult women and the results may not be generalizable to men or to other age groups.

For peer review only - http://bmjopen.bmj.com/site/about/guidelines.xhtml

Introduction

Quality of life (QoL) and mental health are amongst the most important outcomes for individuals, but the prevalence of problems is high [1, 2]. Improving QoL and reducing the mental health burden is challenging but there is consensus that public health strategies should include prevention, timely diagnosis, and optimising management and treatment of prevalent cases [3].

Early diagnosis and treatment of patients with adverse mental health outcomes is not always possible, in part because symptoms are often unspecific and go unrecognised, and because patients do not always seek care for mental health-related conditions [4, 5]. There has been a lack of research quantifying the burden of mental health and other QoL-related complaints that have not been picked up in primary care, and therefore may remain undiagnosed and untreated [6]. One way of quantifying the gap between adverse mental health and QoL-related outcomes recorded in primary care, and those experienced by patients, is to directly collect patient-reported outcomes (PROs) and compare with information on the same outcomes in the clinical record. Under-recording of problems in primary care records could suggest lack of awareness by the general practitioner (GP) about the patient's lack of wellbeing, and thus a missed opportunity for care. Under-recording might also reflect inconsistent coding of mental health and QoL problems in the primary care record, with important implications for audit and research based on electronic health records (EHR) [7, 8].

In this study, we compared patient-reported information on symptoms of anxiety, depression, and QoL domains, with data for similar constructs registered in the patients' EHR. We used data from a previous study that collected PROs data from a convenience sample of women with and without history of breast cancer [9], and for whom EHR data were available.

For peer review only

For peer review only - http://bmjopen.bmj.com/site/about/guidelines.xhtml

Methods

Study design and sampling frame

We used a convenience sample of women with PRO data available from a previous study [9]. For the original study, primary care practices contributing with data to the Clinical Practice Research Datalink (CPRD) GOLD primary care database in August 2018 were invited to participate. CPRD GOLD includes EHR of patients attending general practices in the UK that use Vision software to manage patient's records. Data are entered in the patients' EHR by GPs during consultations using Read codes [10], which include information on symptoms, diagnoses, and prescriptions [11]. The study protocol details provided details on the sample size calculations. Patients registered with primary care practices that accepted to participate were considered potentially eligible for the study.

Patient eligibility criteria, selection and recruitment

A full description of eligibility and recruitment has been published elsewhere [9]. Briefly, inclusion criteria for the breast cancer survivors' group were 1) diagnosis of invasive breast cancer at least one year before (all stages) and 2) aged 18-80 years. To ensure that the recorded breast cancer was incident, we required one year of follow-up in CPRD prior to the breast cancer diagnosis. For the comparison group, inclusion criteria were 1) no history of cancer (except non-melanoma skin cancer), 2) aged 18-80 years, and 3) at least 2 years of follow-up data in CPRD (since we required one year of follow-up before and after cancer to be included in the breast cancer group). Exclusion criteria for both groups were 1) inability to complete a self-reported questionnaire (e.g. due to dementia) and 2) having had another (non-breast) cancer or having been treated for a non-invasive breast tumour.

The CPRD GOLD primary care database was used to identify all breast cancer survivors from participating practices, and a random sample of women with no prior cancer (frequency matched on age to breast cancer survivors) from the same practices. GPs applied inclusion and exclusion criteria (*vide* above), and sent the study materials to the eligible patients' addresses with a pre-paid envelope to return the questionnaires. Patients were recruited between January and November 2019.

Patient-reported outcomes

Anxiety and depression

For peer review only - http://bmjopen.bmj.com/site/about/guidelines.xhtml

Anxiety and depressive symptoms were measured with the Hospital Anxiety and Depression Scale (HADS) [12]. This is a 14-item self-reported screening tool for anxiety and depressive symptoms in the past week [12]. Based on their responses, we categorised patients as non-case (scores 0-7), borderline (scores 8-10) and probable case (scores 11-21) [12].

The QoL impact of anxiety and depression were measured with the respective domains in the Quality of Life in Adult Cancer Survivors Scale (QLACS) (see below).

Quality of life

QoL was assessed with QLACS [13]. This tool includes 47 items, divided in 7 generic domains (i.e. negative feelings; positive feelings; cognitive problems; pain; sexual function/interest; energy/fatigue; and social avoidance), and 5 cancer-specific domains (i.e. financial problems; benefits of cancer; distress-family; appearance; distress-recurrence) which are not considered further in this paper. Of the 7 generic QoL domains, 6 were considered suitable for comparison with data in the EHR because women with distress for these domains may visit their GP to seek help: 'negative feelings', 'cognitive problems', 'pain', 'sexual problems', 'fatigue' and 'social avoidance'. Each domain considered has 4 items on the QLACS questionnaire. Participants are instructed to answer in relation to the previous four weeks. Responses to each item are given on a Likert-type of scale that varies between 1 (never) and 7 (always); higher scores indicate poorer QoL.

To identify women who had high levels of distress for each domain, we calculated the mean response (i.e. the sum of the individual item scores divided by four; mean values range between one and seven). We considered a mean of ≥5 (corresponding to average replies of frequently, very often or always experiencing the stated symptom) to reflect distress in that domain. This was varied in sensitivity analyses (see below).

<u>Outcomes recorded in electronic health records primary care data</u>

We extracted the primary care EHR data for all participants. As PROs were collected between January and November 2019, we extracted data from the January 2020 version of CPRD GOLD, which included data from 1987 up to December 2019.

Anxiety and depression were defined using lists of Read codes from a systematic review [14]. For the QoL domains, we produced lists of Read codes closely related to the items in the QLACS domain (Supplementary Table 1). Read codes were used to identify women with these codes registered in their EHR in the 3, 6, 12 and 24 months prior to the date of last data collection from the practice. The last collection date varied from practice to practice, but was generally within three weeks of the database version (e.g. in the January 2020 version, the

For peer review only - http://bmjopen.bmj.com/site/about/guidelines.xhtml

date of last data collection from the practices was in median 20 days (inter-quartile range: 19-20) prior to 31 December 2019).

Data analysis

We calculated the proportion of women who reported high levels of distress in the questionnaires and had similar information in their EHR (i.e. sensitivity of the EHR for capturing patient-reported distress). To better understand the agreement between PROs and the EHR data, as a secondary analysis, we also calculated the proportion of women with codes indicating distress on each domain in their EHR that reported distress levels in the questionnaires (positive predictive value of the EHR for capturing patient-reported distress). Results were shown in tables and descriptively.

Sensitivity analysis

As we used an arbitrary cut-off to identify patients with poor QoL (mean domain-specific score of ≥5), two sensitivity analyses were conducted: 1) using a lower cut-off of ≥3; 2) considering a score of ≥5 on at least one item in the domain (rather than the mean) as reflecting distress. Finally, we explored whether breast cancer survivors had different results compared to women with no history of cancer.

Patient and Public Involvement

The authors are thankful to the cancer survivors involved with the Independent Cancer Patients' Voice (http://www.independentcancerpatientsvoice.org.uk/), a patient advocate group, for their comments on the study protocol.

For peer review only - http://bmjopen.bmj.com/site/about/guidelines.xhtml

Results

Characteristics of the participants

608 women from 40 primary care practices participated in the study (Table 1). General practices were from all four UK countries, but there was a predominance of practices from Scotland (N=16) and Wales (N=15) (Supplementary Table 2). The median number of consultations in 2018 and 2019 was 11, similar between breast cancer survivors (median 11, inter-quartile range (IQR): 7-16) and women with no history of cancer (median 11, inter-quartile range (IQR): 7-20). A quarter of the women had a higher education degree.

Table 1 Characteristics of the study participants. *

	All participants (N=608)	
	N	%
**Age at completion of questionnaire**		
34-59 years	174	28.6
60-69 years	210	34.5
≥70 years	224	36.8
**Highest education level**		
Up to GCSEs, O levels, or equivalent	205	33.7
A levels or equivalent	65	10.7
Trade or technical training	106	17.4
Undergraduate or post-graduate degree	160	26.3
Did not want to disclose	72	11.8
**Ethnicity**		
White	589	96.9
Asian / Asian British	7	1.2
Did not want to disclose	12	2.0
**IMD quintile**		
1 (least deprived)	124	20.4
2	90	14.8
3	81	13.3
4	239	39.3
5 (most deprived)	74	12.2
**Living arrangements**		
Not alone	458	75.3
Alone	138	22.7
Did not want to disclose	12	2.0
**Country**		
England	114	18.8
Northern Ireland	49	8.1
Scotland	188	30.9
Wales	257	42.3

IMD = Index of Multiple Deprivation

* Proportion may not add to 100% due to rounding

For peer review only

Anxiety and depression

Of the 599 women that replied to the anxiety subscale, 242 (40%) had borderline to abnormal symptoms (Table 2). Borderline to abnormal symptoms of depression were also reported by 92 (15%) of the 601 women that replied to the subscale for depression. Almost no women had Read codes for anxiety or depression registered in their EHR in the 24 months prior. However, 108/242 (45%) of those reporting anxiety symptoms were prescribed with an anxiolytic or antidepressant (for anxiety), and 51/92 (55%) of those reporting depression symptoms were prescribed an antidepressant.

In the QoL scale, 100 of the 608 (17%) women that replied to questionnaire had average replies of frequently, very often or always experiencing negative feelings (mean score ≥5). Only 1 patient (1%) had Read codes related to anxiety and/or depression recorded in their EHR in the 24 months prior, but 51 (51%) had an antidepressant and/or anxiolytic prescription.

Of the patients that had information about negative feelings in their EHR, only a minority reported distress in the questionnaires (Supplementary table 3).

Other QoL domains: Cognitive problems, fatigue, pain, sexual dysfunction, social avoidance

93/608 (16%) women reported high levels of distress related to cognitive problems, 156 (26%) to sexual dysfunction, 157 (26%) to fatigue/energy, and 82 (14%) to social avoidance (Table 3). No codes relevant to these domains were found in the patients' EHR up to 24 months prior. Using lower cut-offs to classify patients based in their QoL scores yielded similar results. Distress with pain was reported in the questionnaires by 123 (21%) of the women, and 65 (53%) of these had symptoms of pain and/or analgesic prescription recorded their EHR in the previous three months; this increased to 92 (75%) when a longer 24-month time window was used.

Sensitivity analyses

Results were similar to those of the main analysis when different criteria were used to define distress (see Table 2 and Table 3). There were no meaningful differences in the results between breast cancer survivors and women with no history of cancer (see Supplementary Table 4 and Supplementary Table 5).

For peer review only - http://bmjopen.bmj.com/site/about/guidelines.xhtml

Table 2 Capture of patient-reported anxiety and depression in patients' primary care records.

Patient reported outcomes			Read codes for symptoms/diagnoses in the patients' electronic health records by time prior to LCD								Relevant drug prescription by time prior to LCD *							
			3mo		6mo		12mo		24mo		3mo		6mo		12mo		24mo	
	N	%	N	%	N	%	N	%	N	%	N	%	N	%	N	%	N	%
**HADS**																		
**Anxiety**																		
Normal	357	59.6	0	0	0	0	0	0	0	0	53	14.9	55	15.4	67	18.8	84	23.5
Borderline	124	19.7	0	0	0	0	0	0	0	0	33	28.0	34	33.9	40	33.9	49	41.5
Abnormal	118	20.7	1	0.8	1	0.8	2	1.6	2	1.6	40	32.3	42	28.8	53	42.7	59	50.0
**Depression**																		
Normal	509	84.7	0		0		0		0		75	14.7	77	15.1	96	18.9	120	23.6
Borderline	66	4.3	0		0		0		0		26	39.4	26	39.4	30	45.5	34	51.5
Abnormal	26	11.0	0		0		0		0		13	50.0	13	50.0	16	61.5	17	65.4
**QLACS**																		
**Negative feelings**																		
≥5	100	16.8	1	1.0	1	1.0	1	1.0	1	1.0	36	36.0	37	37.0	47	47.0	51	51.0
≥3	386	64.7	1	0.3	1	0.3	2	0.5	2	0.5	89	23.1	90	23.3	109	28.2	129	33.4
1 item ≥5	227	37.3	1	0.4	1	0.4	1	0.4	1	0.4	65	28.6	66	29.1	82	36.1	95	41.9

PRO = Patient reported outcomes; HADS = Hospital Anxiety and Depression Scale; QLACS = Quality of Life in Adults Cancer Survivors Scale. LCD = last collection date for the practice; mo = months. * Anxiolytics or antidepressants for anxiety; antidepressants for depression.

For peer review only

Table 3 Capture of patient-reported QoL-related distress in patients' primary care records.

QoL Domain	Domain score	Patient reported outcomes		Relevant Read codes in the electronic health record*, by time prior to the date of last data collection							
				3mo		6mo		12mo		24mo	
		No. §	%	No.	%	No.	%	No.	%	No.	%
Cognitive problems	Average ≥5	93	15.5	0		0		0		0	
	Average ≥3	394	65.6	0		0		0		0	
	1 item ≥5	193	31.7	0		0		0		0	
Fatigue	Average ≥5	157	26.1	0		0		0		0	
	Average ≥3	472	78.5	0		0		0		0	
	1 item ≥5	536	88.2	0		0		0		0	
Physical pain	Average ≥5	123	20.6	65	52.8	70	56.9	82	66.7	92	74.8
	Average ≥3	330	55.4	106	32.1	116	35.2	152	46.1	186	56.4
	1 item ≥5	231	38.0	86	37.2	94	40.7	120	52.0	142	61.5
Sexual dysfunction	Average ≥5	156	25.7	0		0		0		0	
	Average ≥3	377	62.0	0		0		0		0	
	1 item ≥5	304	50.0	0		0		0		0	
Social avoidance	Average ≥5	82	13.5	0		0		0		0	
	Average ≥3	294	48.4	0		0		0		0	
	1 item ≥5	196	32.2	0		0		0		0	

QoL = quality of life; mo = months. * Severe cognitive dysfunction was an exclusion criterion for the study. § 608 women participated in the study; due to missing data for some items, the number of women included in the denominator varies slightly by domain. *Relevant Read codes were codes for cognitive impairment, dementia and dementia specific drugs (cognitive problems domain); low energy, tiredness (fatigue domain); pain, pain syndromes, analgesics prescriptions (pain domain); low libido, anorgasm, vaginismus (sexual dysfunction); social isolation and avoidance (social avoidance domain).

For peer review only

Discussion

Summary

Most patients who reported clinically relevant symptoms of anxiety and depression, and distress with cognitive problems, fatigue, physical pain, sexual dysfunction and social avoidance, did not have clinical codes for these conditions in their primary care EHR. This suggests that in some cases GPs may be unaware of problems adversely affecting their patients' QoL. Our results may also be partly explained by inconsistent coding, as evident from the number of women in receipt of medications for anxiety and depression, despite no diagnostic codes being present in the EHR. In these cases, GPs were evidently aware of the patient's condition but had not entered a diagnostic code into the EHR, which could lead to misleading information when routine coded data are used for audit and research.

Strengths and limitations

The ability to compare PROs with data available in the EHR represents a unique strength of this study. To our knowledge, no previous study has reported on this comparison. However, this study had limitations. We did not collect data on the date of questionnaire completion or questionnaire return to the research team, and therefore we could identify, precisely, the consultations that corresponded to when the PROs were evaluated. As questionnaires were returned over a 9-month period, this could have affected our assessment of outcomes particularly in 3 to 6 months prior. However, even analyses looking at coding in the previous 24 months showed substantial under-recording of mental-health and QoL-related distress in coded primary care data. Our approach to identify patients experiencing distress on specific QLACS domains used score thresholds that were not validated. However, sensitivity analysis using different cut-offs showed generally the same patterns. CPRD only captures drugs prescribed to patients, and widely used drugs for pain and fatigue are sold over-the-counter. We assumed that anxiolytics and antidepressants were taken for anxiety/depression, but we cannot rule out that some were for other indications such as pain or insomnia. The comparison for cognitive problems was limited by the need to exclude patients unable to reply to a self-reported questionnaire, and we cannot rule out that GPs may have been overly strict in applying this exclusion criterion, excluding mild cognitive impairment. Our results are based on a convenience sample of breast cancer survivors and non-cancer controls and may not be generalisable to the general population; however, results were similar in our sensitivity analysis comparing results between the two groups, which is probably because women with history of breast cancer were on average 8 years post diagnosis and most likely not under active treatment for cancer. Half of the patients in our sample had history of breast cancer, which may have been associated with closer monitoring, and therefore we could have

For peer review only - http://bmjopen.bmj.com/site/about/guidelines.xhtml

underestimated the extent of the missed coding of these problems. However, four sensitivity analyses comparing those with and without prior cancer showed no major differences between groups. We compared PROs with information coded in the EHR; while most of patient care is coded using records, GPs sometimes use other methods to keep records (e.g. free text entry) which are not available to us. Similarly, some codes in the EHR are unspecific (e.g. mood observations) and we could not assign a correspondence to domains of QoL. Ford et al. [15] explored the reasons for differences in coding for mental health conditions in primary care, and found that GPs may prefer free text and use codes for symptoms or general codes instead of definitive diagnoses. Therefore, it is possible that we underestimated, in some cases, the awareness of the GP about the patients' wellbeing.

Comparison with existing literature

Only one in 3 patients that reported distressing levels of negative feelings had similar information recorded in their EHR in the previous 3 months. This is consistent with patients often not seeking primary care for anxiety and depression [16]. Approximately one-half of the women that reported poor QoL related to pain had related information in the EHR in the previous 3 months. This may be partly explained by patients self-treating pain with widely used over-the-counter treatments such as paracetamol and ibuprofen. Conversely, the higher recording of pain compared to negative feelings could be related to patients more often seeking help for concerns perceived as being amenable to treatment. Patients with a prescription of antidepressants / anxiolytics and that reported normal levels of depressive / anxiety symptoms are not unexpected – these drugs are effective at improving symptoms of depression and anxiety, but have long treatment courses and patients are recommended to continue pharmacological treatment for months after symptoms disappear to prevent relapse [17, 18].

We did not find any records of cognitive dysfunction, social avoidance, sexual dysfunction or fatigue in the EHR of the participating women in the previous 24 months. An absence of entries for social avoidance is plausible; Read codes for social avoidance have seldom been used in the database. A lack of records for sexual dysfunction is in keeping with evidence that only a small proportion of people contact GPs for issues related to sexual function [19]. The lack of coded records for cognitive dysfunction and fatigue was more unexpected. It is possible that GPs systematically excluded people with mild cognitive dysfunction. For fatigue, a manual review of all entries in the EHR of patients that reported distressing levels of fatigue revealed a pattern of multimorbidity, almost always with diagnoses where fatigue is implicit (e.g., heart failure, chronic obstructive pulmonary disease), but no explicit codes for fatigue.

For peer review only - http://bmjopen.bmj.com/site/about/guidelines.xhtml

Implications for research and/or practice

It is important to raise awareness that patients may not always actively report their distress. Even when GPs are aware of health issues, they are not always coded in the patient record, and thus EHRs have low sensitivity to detect patients experiencing poor QoL at a particular point in time. Studies investigating anxiety and depression should consider prescriptions as well as clinical codes, as many patients were prescribed anxiolytics and antidepressants without having a Read code for these conditions.

Similarly to other studies [20], in this study the collection of PRO data was not, unfortunately, followed by feedback of the results to the patients or to the patients' GPs. This was because the authorisation to conduct this study within the UK National Health Service was granted on the basis that there would separation between the researchers and the identity of patients and GPs, and we could only access anonymised data. Krageloh et al., 2015 [20] highlight in their review that most studies where there was a formal procedure to feedback PRO results to patients and health care providers reported better outcomes in this group compared to controls [20]. Future studies of PRO outcomes in the NHS should explore options to report back results without violating the data protection regulation in place.

Conclusion

We found substantial under-recording of mental-health and QoL-related distress in coded primary care data. In addition, there may be inconsistent coding of known conditions, meaning that studies of mental-health and QoL-related outcomes using EHR databases likely underestimate the absolute burden of these outcomes in the population. Further research is needed to understand whether or not GPs are aware of patient distress in cases where codes have not been recorded.

For peer review only - http://bmjopen.bmj.com/site/about/guidelines.xhtml

Contributorship Statement

HC, RW, and KB designed the study. HC, RW, and KB obtained ethical approvals. HD, HC, RW, and KB managed data collection. HC entered the data and performed analyses. All authors revised the manuscript for important intellectual content and approved the final version of the manuscript.

Funding and role of funding

H.C. was supported by an MRC Case Studentship in Epidemiology, jointly funded by UK Medical Research Council (MRC; https://mrc.ukri.org/) and the Clinical Practice Research Datalink (https://www.cprd.com/) at the Medicines and Healthcare Products Regulatory Agency (grant number MR/M016234/1 to H.C.). K.B. is funded by a Wellcome Senior Research Fellowship (grant number 220283/Z/20/Z) and previously held a Sir Henry Dale Fellowship jointly funded by Wellcome Trust (https://wellcome.ac.uk/) and the Royal Society (https://royalsociety.org/) (grant number 107737/Z/15/Z). The funders had no role in study design, data collection and analysis, decision to publish, or preparation of the manuscript.

Competing interests

Dr Bhaskaran reports grants from Wellcome Trust, the Royal Society, Medical Research Council, and British Heart Foundation, outside the submitted work. Dr Williams and Ms Dempsey report that CPRD has financial relationships with its clients, including the London School of Hygiene and Tropical Medicine, in relation to providing access to research data and services outside the submitted work. HC has no conflict of interest to disclose.

Ethics approval and consent to participate

This study was approved by the East of England - Cambridge South Research Ethics Committee (Ref: 17/EE/0403), the London School of Hygiene and Tropical Medicine Interventions Research Ethics Committee (Ref: 14417) and the Health Research Authority and Health and Care Research Wales (IRAS Project ID: 224561). Implicit patient consent was obtained when the patient posted the completed questionnaires.

For peer review only

Data sharing statement

This study is based in part on data from the Clinical Practice Research Datalink obtained under licence from the UK Medicines and Healthcare products Regulatory Agency. The terms of our licence to access the data preclude us from sharing individual patient data with third parties. The electronic health records raw data may be requested directly from CPRD following their usual procedures.

Open Access

This research was funded in part by the Wellcome Trust [grant numbers 107737/Z/15/Z and 220283/Z/20/Z]. For the purpose of open access, the authors have applied a CC BY public copyright licence to any Author Accepted Manuscript version arising from this submission.

For peer review only

For peer review only - http://bmjopen.bmj.com/site/about/guidelines.xhtml

References

1. *Quality of Life - The assessment, analysis and reporting of patient-reported outcomes*. third ed. 2016, UK: John Wiley & Sons, Ltd.
2. Institute for Health Metrics and Evaluation. *GBD Compare: Years Lived with Disability, United Kingdom, 2019*. [cited 2021; Available from: https://vizhub.healthdata.org/gbd-compare/.
3. Patel, V., et al., *The Lancet Commission on global mental health and sustainable development.* Lancet, 2018. **392**(10157): p. 1553-1598.
4. Campion, J., et al., *Public mental health: required actions to address implementation failure in the context of COVID-19.* Lancet Psychiatry, 2022. **9**(2): p. 169-182.
5. Alonso, J., et al., *Treatment gap for anxiety disorders is global: Results of the World Mental Health Surveys in 21 countries.* Depress Anxiety, 2018. **35**(3): p. 195-208.
6. National Institute for Health and Care Excellence (NICE), *Common mental health problems: identification and pathways to care. Clinical guideline 123*. 2011.
7. Gray, J., D. Orr, and A. Majeed, *Use of Read codes in diabetes management in a south London primary care group: implications for establishing disease registers.* BMJ, 2003. **326**(7399): p. 1130.
8. Shephard, E., S. Stapley, and W. Hamilton, *The use of electronic databases in primary care research.* Fam Pract, 2011. **28**(4): p. 352-4.
9. Carreira, H., et al., *Quality of life and mental health in breast cancer survivors compared with non-cancer controls: a study of patient-reported outcomes in the United Kingdom.* J Cancer Surviv, 2021. **15**(4): p. 564-575.
10. Chisholm, J., *The Read clinical classification.* BMJ, 1990. **300**(6732): p. 1092.
11. Herrett, E., et al., *Data Resource Profile: Clinical Practice Research Datalink (CPRD).* Int J Epidemiol, 2015. **44**(3): p. 827-36.
12. Zigmond, A.S. and R.P. Snaith, *The hospital anxiety and depression scale.* Acta Psychiatr Scand, 1983. **67**(6): p. 361-70.
13. Avis, N.E., et al., *Assessing quality of life in adult cancer survivors (QLACS).* Qual Life Res, 2005. **14**(4): p. 1007-23.
14. Carreira, H., et al., *Identification of mental health and quality of life outcomes in primary care databases in the UK: a systematic review.* BMJ Open, 2019. **9**(7): p. e029227.
15. Ford, E., et al., *"You don't immediately stick a label on them": a qualitative study of influences on general practitioners' recording of anxiety disorders.* BMJ Open, 2016. **6**(6): p. e010746.
16. Clement, S., et al., *What is the impact of mental health-related stigma on help-seeking? A systematic review of quantitative and qualitative studies.* Psychol Med, 2015. **45**(1): p. 11-27.
17. National Institute for Health and Care Excellence (NICE), *Depression in adults: treatment and management. Clinical Guideline 222*. 2022.
18. National Institute for Health and Care Excellence (NICE), *Generalised anxiety disorder and panic disorder in adults: management (CG113)*. 2011.
19. Hinchliff, S., et al., *Seeking help for sexual difficulties: findings from a study with older adults in four European countries.* Eur J Ageing, 2020. **17**(2): p. 185-195.
20. Krägeloh, C.U., et al., *Using Feedback From Patient-Reported Outcome Measures in Mental Health Services: A Scoping Study and Typology.* Psychiatric Services, 2015. **66**(3): p. 224-241.

For peer review only - http://bmjopen.bmj.com/site/about/guidelines.xhtml

Supplementary Table 1 Matching between QoL domain and information in the EHRs.

QoL domain	QLACS Items	Search in the EHR for Read codes* related to:
Negative feelings	19 Bothered by mood swings<br>7 Felt blue or depressed<br>9 Worried about little things<br>24 Felt anxious	Depression and/or anxiety (disorders and symptoms), antidepressants, or anxiolytic prescription
Cognitive problems	3 Bothered by having a short attention span<br>4 Had trouble remembering things<br>2 Difficulty doing things requiring concentration<br>23 Bothered by forgetting what started to do	Cognitive impairment; cognitive dysfunction symptoms; dementia*; dementia-specific drug*.
Physical pain	13 Bothered by pain preventing activities<br>17 Mood disrupted by pain or its treatment<br>27 Pain interfered with social activities<br>21 Had aches or pains	Pain; painful conditions; prescriptions of analgesics.
Sexual problems	16 Lacked interest in sex<br>26 Avoided sexual activity<br>12 Dissatisfied with sex life<br>10 Bothered by inability to function sexually	Low libido; anorgasmia; vaginismus.
Fatigue	11 Lacked energy to do things wanted to<br>14 Felt tired a lot<br>1 Had energy to do things wanted to do<br>5 Felt fatigued	Low energy; tiredness.
Social avoidance	18 Avoided social gatherings<br>20 Avoided friends<br>25 Reluctant to meet new people<br>15 Reluctant to start new relationships	Social isolation, or social avoidance.

* Definitions were based on a comprehensive systematic review of the studies that defined mental health and quality of life-related outcomes in primary care databases of electronic health records [11].

QLACS = Quality of Life in Adult Cancer Survivors Scale; EHR = electronic health records

For peer review only

Supplementary Table 2 Characteristics of the practices that participated in the study.

	Number of practices in this study (N=40)	Number of practices in CPRD (N=971)
**Country**		
England	6 (15%)	573 (59%)
North West	2 (5.0%)	87 (9.0%)
London	1 (2.5%)	105 (10.8%)
South East	3 (7.5%)	73 (7.5%)
Northern Ireland	3 (7.5%)	42 (4.3%)
Scotland	16 (40.0%)	229 (23.6%)
Wales	15 (37.5%)	127 (13.1%)

For peer review only

Supplementary Table 3 Patient reported outcomes of women with codes related to distress in their primary care record.

			Patients with info for the domain in EHR		Patients scoring as distressed, according to patient-reported data					
					≥5		≥3		At least one item 5	
**Domain**	**Read codes related to:**	**Time prior to LDC**	**No. §**	**%**	**No.**	**%**	**No.**	**%**	**No.**	**%**
Negative feelings	Depression and/or anxiety (disorders and symptoms), antidepressants, or anxiolytic prescription.	3 mo.	118	19.4	36	31.3	89	77.4	65	55.1
		6 mo.	120	19.7	37	31.6	90	76.9	66	55.0
		12 mo.	146	24.0	47	33.1	109	76.8	82	56.2
		24 mo.	170	28.5	51	30.0	129	75.9	95	54.3
Cognitive problems	Cognitive impairment; cognitive dysfunction symptoms; dementia; dementia-specific drug*	3 mo.	0		-		-		-	
		6 mo.	0		-		-		-	
		12 mo.	0		-		-		-	
		24 mo.	0		-		-		-	
Fatigue	Low energy; tiredness.	3 mo.	0		-		-		-	
		6 mo.	0		-		-		-	
		12 mo.	0		-		-		-	
		24 mo.	0		-		-		-	
Physical pain	Pain; painful conditions; prescriptions of analgesics.	3 mo.	138	37.2	65	48.2	106	78.5	86	62.3
		6 mo.	150	24.7	70	47.6	116	78.9	94	62.7
		12 mo.	203	34.1	82	40.4	152	74.9	120	58.0
		24 mo.	264	43.4	92	35.5	186	71.8	142	53.8
Sexual dysfunction	Low libido; anorgasm; vaginismus.	3 mo.	0		-		-		-	
		6 mo.	0		-		-		-	
		12 mo.	0		-		-		-	
		24 mo.	0		-		-		-	
Social avoidance	Social isolation; social avoidance.	3 mo.	0		-		-		-	
		6 mo.	0		-		-		-	
		12 mo.	0		-		-		-	
		24 mo.	0		-		-		-	

EHR = electronic health records; QoL = quality of life; mo = months. * Severe cognitive dysfunction was an exclusion criterion for the study. § 608 women participated in the study; due to missing data for some items, the number of women included in the denominator varies slightly by domain.

For peer review only

Supplementary Table 4 Patient-reported outcomes from women with history of breast cancer and non-cancer controls.

			PROs				Patients scoring above a given threshold in the PRO study that had domain-related information in EHRs, by time prior to the last data collection for the practice																
		Mean domain cut-off	Breast cancer survivors		Non-cancer controls		Breast cancer survivors								Non-cancer controls								
							3mo		6mo		12mo		24mo		3mo		6mo		12mo		24mo		
**QLACS Domain**	**Read codes for**		**No. §**	**%**	**No. §**	**%**	**No.**	**%**	**No.**	**%**	**No.**	**%**	**No.**	**%**	**No.**	**%**	**No.**	**%**	**No.**	**%**	**No.**	**%**	
Negative feelings	Depression and/or anxiety (inc. symptoms), antidepressants, or anxiolytic prescription	≥5	62	17.4	38	15.1	23	37.1	23	37.1	29	46.8	32	51.6	13	34.2	14	36.8	18	47.4	19	50.0	
		≥3	231	64.9	155	61.5	58	25.1	58	25.1	70	30.3	82	35.5	31	20.0	32	20.7	39	25.2	47	30.3	
		1 item ≥5	140	39.3	87	34.5	42	30.0	42	30.0	53	37.9	61	43.6	23	26.4	24	27.6	29	33.3	34	39.1	
Cognitive problems	Cognitive dysfunction; dementia; dementia-specific drug.*	≥5	61	17.1	32	12.7	0		0		0		0		0		0		0		0		
		≥3	238	66.9	156	61.9	0		0		0		0		0		0		0		0		
		1 item ≥5	123	34.6	70	27.8	0		0		0		0		0		0		0		0		
Physical pain	Pain; painful conditions; prescriptions of analgesics.	≥5	72	20.2	51	20.2	65	52.9	70	56.9	82	66.7	92	74.8	28	54.9	30	58.8	37	72.6	40	78.4	
		≥3	194	54.5	136	54.0	106	32.1	116	35.2	152	46.1	186	56.4	42	30.9	45	33.1	65	47.8	77	56.6	
		1 item ≥5	144	40.5	87	34.5	86	37.2	94	40.7	120	52.0	142	61.5	33	37.9	36	41.4	47	54.0	52	59.8	
Sexual dysfunction	Low libido; anorgasm; vaginismus.	≥5	107	30.1	49	19.4	0		0		0		0		0		0		0		0		
		≥3	234	65.7	143	56.8	0		0		0		0		0		0		0		0		
		1 item ≥5	196	55.1	108	42.9	0		0		0		0		0		0		0		0		
Fatigue	Low energy; tiredness.	≥5	104	29.2	53	21.0	0		0		0		0		0		0		0		0		
		≥3	283	79.5	189	75.0	0		0		0		0		0		0		0		0		
		1 item ≥5	318	89.3	218	86.5	0		0		0		0		0		0		0		0		
Social avoidance	Social isolation; social avoidance.	≥5	50	14.0	32	12.7	0		0		0		0		0		0		0		0		
		≥3	171	48.0	123	48.8	0		0		0		0		0		0		0		0		
		1 item ≥5	125	35.1	71	28.2	0		0		0		0		0		0		0		0		

EHR = electronic health records; QoL = Health-Related Quality of Life; mo. = month. * Severe cognitive dysfunction was an exclusion criterion for the study. § 608 women participated in the study; due to missing data for some items, the number of women included in the denominator varies slightly by domain.

For peer review only

For peer review only - http://bmjopen.bmj.com/site/about/guidelines.xhtml

Supplementary Table 5 Patient-reported outcomes of patients with codes related to distress in their primary care record, by history of breast cancer.

			Patients with info for the domain in EHR				Patients scoring as distressed, according to patient-reported data												
							Breast cancer survivors						Non-cancer controls					
			Breast cancer survivors		Non-cancer controls		≥5		≥3		At least one item 5		≥5		≥3		At least one item 5	
**Domain**	**Read codes related to:**	**Time prior to LDC**	**No. §**	**%**	**No. §**	**%**	**No.**	**%**	**No.**	**%**	**No.**	**%**	**No.**	**%**	**No.**	**%**	**No.**	**%**
Negative feelings	Depression and/or anxiety (disorders and symptoms), antidepressants, or anxiolytics.	3 mo.	75	21.7	40	15.9	23	30.7	58	77.3	42	54.6	13	32.5	31	77.5	23	56.1
		6 mo.	76	22.0	41	16.3	23	30.3	58	76.3	42	53.9	14	34.2	32	78.1	24	57.1
		12 mo.	92	26.6	50	19.9	29	31.5	70	76.1	53	55.8	18	36.0	39	78.0	29	33.3
		24 mo.	109	31.5	61	24.3	32	29.4	82	75.2	61	54.0	19	31.2	47	77.1	34	54.8
Cognitive problems	Cognitive impairment; dementia; specific drugs*	3 mo.	0				-		-		-		-		-		-	
		6 mo.	0				-		-		-		-		-		-	
		12 mo.	0				-		-		-		-		-		-	
		24 mo.	0				-		-		-		-		-		-	
Physical pain	Pain; painful conditions; prescriptions of analgesics.	3 mo.	89	25.0	49	19.4	37	41.6	64	71.9	53	59.6	28	57.1	42	85.7	33	67.4
		6 mo.	98	27.5	52	20.6	40	40.8	71	72.5	58	59.2	30	57.7	45	86.5	36	69.2
		12 mo.	127	35.7	80	31.8	45	35.4	87	68.5	73	50.7	37	46.3	65	81.3	47	58.8
		24 mo.	162	45.5	102	40.5	52	32.1	109	67.3	90	55.6	40	39.2	77	75.5	52	51.0
Sexual dysfunction	Low libido; anorgasm; vaginismus.	3 mo.	0				-		-		-		-		-		-	
		6 mo.	0				-		-		-		-		-		-	
		12 mo.	0				-		-		-		-		-		-	
		24 mo.	0				-		-		-		-		-		-	
Fatigue	Low energy; tiredness.	3 mo.	0				-		-		-		-		-		-	
		6 mo.	0				-		-		-		-		-		-	
		12 mo.	0				-		-		-		-		-		-	
		24 mo.	0				-		-		-		-		-		-	
Social avoidance	Social isolation; social avoidance.	3 mo.	0				-		-		-		-		-		-	
		6 mo.	0				-		-		-		-		-		-	
		12 mo.	0				-		-		-		-		-		-	
		24 mo.	0				-		-		-		-		-		-	

EHR = electronic health records; QoL = Health-Related Quality of Life; mo. = month. * Severe cognitive dysfunction was an exclusion criterion for the study. § 608 women participated in the study; due to missing data for some items, the number of women included in the denominator varies slightly by domain.

For peer review only

STROBE Statement—Checklist of items that should be included in reports of ***cross-sectional studies***

	Item No	Recommendation	Location of information in the manuscript
**Title and abstract**	1	(*a*) Indicate the study's design with a commonly used term in the title or the abstract	Done, see title.
		(*b*) Provide in the abstract an informative and balanced summary of what was done and what was found	Done, see abstract.
**Introduction**			
Background/rationale	2	Explain the scientific background and rationale for the investigation being reported	p. 5
Objectives	3	State specific objectives, including any prespecified hypotheses	p. 5, lines 20-23
**Methods**			
Study design	4	Present key elements of study design early in the paper	p. 6, lines 2-10
Setting	5	Describe the setting, locations, and relevant dates, including periods of recruitment, exposure, follow-up, and data collection	p. 6, lines 2-28
Participants	6	(*a*) Give the eligibility criteria, and the sources and methods of selection of participants	p. 6, lines 12-22
Variables	7	Clearly define all outcomes, exposures, predictors, potential confounders, and effect modifiers. Give diagnostic criteria, if applicable	p. 7
Data sources/ measurement	8*	For each variable of interest, give sources of data and details of methods of assessment (measurement). Describe comparability of assessment methods if there is more than one group	pp. 6-8
Bias	9	Describe any efforts to address potential sources of bias	p. 7
Study size	10	Explain how the study size was arrived at	p. 6
Quantitative variables	11	Explain how quantitative variables were handled in the analyses. If applicable, describe which groupings were chosen and why	p. 8, lines 3-16
Statistical methods	12	(*a*) Describe all statistical methods, including those used to control for confounding	p. 8, lines 3-16
		(*b*) Describe any methods used to examine subgroups and interactions	p. 8, lines 3-16
		(*c*) Explain how missing data were addressed	p. 8
		(*d*) If applicable, describe analytical methods taking account of sampling strategy	n/a
		(*e*) Describe any sensitivity analyses	p. 8, lines 18-21
**Results**			
Participants	13*	(a) Report numbers of individuals at each stage of study—eg numbers potentially eligible, examined for eligibility, confirmed eligible, included in the study, completing follow-up, and analysed	p. 6, line 12
		(b) Give reasons for non-participation at each stage	p. 6, line 12
		(c) Consider use of a flow diagram	n/a

For peer review only

Descriptive data	14*	(a) Give characteristics of study participants (eg demographic, clinical, social) and information on exposures and potential confounders	Table 1.
		(b) Indicate number of participants with missing data for each variable of interest	Table 1.
Outcome data	15*	Report numbers of outcome events or summary measures	Tables 2 and 3.
Main results	16	(*a*) Give unadjusted estimates and, if applicable, confounder-adjusted estimates and their precision (eg, 95% confidence interval). Make clear which confounders were adjusted for and why they were included	n/a
		(*b*) Report category boundaries when continuous variables were categorized	n/a
		(*c*) If relevant, consider translating estimates of relative risk into absolute risk for a meaningful time period	n/a
Other analyses	17	Report other analyses done—eg analyses of subgroups and interactions, and sensitivity analyses	p. 10, lines 24-28
**Discussion**			
Key results	18	Summarise key results with reference to study objectives	p. 13, lines 2-11
Limitations	19	Discuss limitations of the study, taking into account sources of potential bias or imprecision. Discuss both direction and magnitude of any potential bias	p. 13, lines 12-35
Interpretation	20	Give a cautious overall interpretation of results considering objectives, limitations, multiplicity of analyses, results from similar studies, and other relevant evidence	p. 14, lines 1-19
Generalisability	21	Discuss the generalisability (external validity) of the study results	p.13, lines 27-29
**Other information**			
Funding	22	Give the source of funding and the role of the funders for the present study and, if applicable, for the original study on which the present article is based	p. 15

*Give information separately for exposed and unexposed groups.

Note: An Explanation and Elaboration article discusses each checklist item and gives methodological background and published examples of transparent reporting. The STROBE checklist is best used in conjunction with this article (freely available on the Web sites of PLoS Medicine at http://www.plosmedicine.org/, Annals of Internal Medicine at http://www.annals.org/, and Epidemiology at http://www.epidem.com/). Information on the STROBE Initiative is available at www.strobe-statement.org.

For peer review only

For peer review only - http://bmjopen.bmj.com/site/about/guidelines.xhtml

BMJ Open

Recording of patients' mental health and quality of life-related outcomes in primary care: a cross-sectional study in the UK

Journal:	*BMJ Open*
Manuscript ID	bmjopen-2022-066949.R2
Article Type:	Original research
Date Submitted by the Author:	17-Nov-2022
Complete List of Authors:	Carreira, Helena; London School of Hygiene & Tropical Medicine, Department of Non-Communicable Disease Epidemiology<br>Williams, Rachael; Clinical Practice Research Datalink<br>Dempsey, Harley; Medicines and Healthcare Products Regulatory Agency<br>Bhaskaran, Krishnan; LSHTM, NCDE
**Primary Subject Heading**:	General practice / Family practice
Secondary Subject Heading:	Epidemiology
Keywords:	PRIMARY CARE, MENTAL HEALTH, GENERAL MEDICINE (see Internal Medicine)

SCHOLARONE™
Manuscripts

I, the Submitting Author has the right to grant and does grant on behalf of all authors of the Work (as defined in the below author licence), an exclusive licence and/or a non-exclusive licence for contributions from authors who are: i) UK Crown employees; ii) where BMJ has agreed a CC-BY licence shall apply, and/or iii) in accordance with the terms applicable for US Federal Government officers or employees acting as part of their official duties; on a worldwide, perpetual, irrevocable, royalty-free basis to BMJ Publishing Group Ltd ("BMJ") its licensees and where the relevant Journal is co-owned by BMJ to the co-owners of the Journal, to publish the Work in this journal and any other BMJ products and to exploit all rights, as set out in our licence.

The Submitting Author accepts and understands that any supply made under these terms is made by BMJ to the Submitting Author unless you are acting as an employee on behalf of your employer or a postgraduate student of an affiliated institution which is paying any applicable article publishing charge ("APC") for Open Access articles. Where the Submitting Author wishes to make the Work available on an Open Access basis (and intends to pay the relevant APC), the terms of reuse of such Open Access shall be governed by a Creative Commons licence – details of these licences and which Creative Commons licence will apply to this Work are set out in our licence referred to above.

Other than as permitted in any relevant BMJ Author's Self Archiving Policies, I confirm this Work has not been accepted for publication elsewhere, is not being considered for publication elsewhere and does not duplicate material already published. I confirm all authors consent to publication of this Work and authorise the granting of this licence.

For peer review only

Recording of patients' mental health and quality of life-related outcomes in primary care: a cross-sectional study in the UK

Helena Carreira, MSc PhD, [1] Rachael Williams, MSc PhD,[2] Harley Dempsey, MSc,[3] Krishnan Bhaskaran, MSc PhD[4]

Authors' affiliations:

[1] Assistant Professor, Department of Non-Communicable Disease Epidemiology, Faculty of Epidemiology and Population Health, London School of Hygiene & Tropical Medicine, Keppel Street, London WC1E 7HT, United Kingdom. https://orcid.org/0000-0003-1538-2526

[2] Observational Research Team Leader, Clinical Practice Research Datalink (CPRD), Medicines and Healthcare products Regulatory Agency, 10th Floor, 10 South Colonnade, Canary Wharf, London E14 4PU, United Kingdom. https://orcid.org/0000-0001-9007-6292

[3] Clinical Study Manager, Clinical Practice Research Datalink (CPRD), Medicines and Healthcare products Regulatory Agency, 10th Floor, 10 South Colonnade, Canary Wharf, London E14 4PU, United Kingdom. https://orcid.org/0000-0001-9605-1264

[4] Professor, Department of Non-Communicable Disease Epidemiology, Faculty of Epidemiology and Population Health, London School of Hygiene & Tropical Medicine, Keppel Street, London WC1E 7HT, United Kingdom. https://orcid.org/0000-0001-5364-8757

Correspondence to:

Helena Carreira

Department of Non-communicable Disease Epidemiology

London School of Hygiene and Tropical Medicine

Keppel Street, London, WC1E 7HT, UK

helena.carreira@lshtm.ac.uk

Keywords: anxiety, depression, quality of life, electronic health records, primary care, UK.

For peer review only

Abstract

Objective: To compare patient-reported anxiety, depression, and quality-of-life (QoL) outcomes, with data registered in patients' primary care electronic health record (EHR).

Design: Cross-sectional study.

Setting: Primary care in the UK.

Participants: 608 women registered in the Clinical Practice Research Datalink (CPRD) GOLD primary care database (a convenience sample using data from a previous study on 356 breast cancer survivors (8.1 years post diagnosis) and 252 women with no prior cancer).

Outcome measures: Patient-reported data on anxiety, depression, and QoL, collected through postal questionnaires, and compared with coded information in EHR up to 2 years prior.

Results: Abnormal anxiety symptoms were reported by 118 of 599 who answered the relevant questions (21%); 59/118 (50%) had GP-recorded anxiolytic/antidepressant use, and 2 (1.6%) had anxiety coded in the EHR. 26/601 women (11%) reported depression symptoms, of whom 17 (65.4%) had GP-recorded antidepressant use and none had depression coded. 65 of 123 women reporting distress on the pain QoL domain (52.8%) had a corresponding record in the EHR <3 months before and 92 (74.8%) <24 months before. No patients reporting fatigue (n=157), sexual health problems (156), social avoidance (82) or cognitive problems (93) had corresponding codes in the EHR. There were no meaningful differences between breast cancer survivors and women with no prior history of cancer.

Conclusion: Many patients reporting mental health and QoL problems had no record of this in coded primary care data. This finding suggests that coded data does not fully reflect the burden of disease. Further research is needed to understand whether or not GPs are aware of patient distress in cases where codes have not been recorded.

Keywords: mental health, quality of life, primary health care, United Kingdom

For peer review only

Strengths and limitations of this study

- A strength of this study comes from the use of the Clinical Practice Research Datalink GOLD primary care database to select participants for the study, as it enabled the comparison of patient-reported outcomes with the data that had been routinely recorded in their electronic health record.
- Patient-reported outcomes were assessed using validated tools and identification of data in the coded electronic health records was based in a systematic review of Read codes.
- Limitations of this study include the lack of information for drugs sold over the counter, which are not captured by CPRD, and that we could not distinguish when anxiolytics and antidepressants may have been used for conditions other than anxiety/depression.
- Most patient care is recorded using codes but General Practitioners sometimes use other methods to keep records (e.g., free text entry) which are not available to us; similarly, some codes in the patient records are unspecific (e.g., mood observations) and we could not assign a correspondence to domains of QoL.
- This study included only a convenience sample of adult women and the results may not be generalizable to all women, men or to other age groups.

For peer review only

For peer review only - http://bmjopen.bmj.com/site/about/guidelines.xhtml

Introduction

Quality of life (QoL) and mental health are amongst the most important outcomes for individuals, but the prevalence of problems is high [1, 2]. Improving QoL and reducing the mental health burden is challenging but there is consensus that public health strategies should include prevention, timely diagnosis, and optimising management and treatment of prevalent cases [3].

Early diagnosis and treatment of patients with adverse mental health outcomes is not always possible, in part because symptoms are often unspecific and go unrecognised, and because patients do not always seek care for mental health-related conditions [4, 5]. There has been a lack of research quantifying the burden of mental health and other QoL-related complaints that have not been picked up in primary care, and therefore may remain undiagnosed and untreated [6]. One way of quantifying the gap between adverse mental health and QoL-related outcomes recorded in primary care, and those experienced by patients, is to directly collect patient-reported outcomes (PROs) and compare with information on the same outcomes in the clinical record. Under-recording of problems in primary care records could suggest lack of awareness by the general practitioner (GP) about the patient's lack of wellbeing, and thus a missed opportunity for care. Under-recording might also reflect inconsistent coding of mental health and QoL problems in the primary care record, with important implications for audit and research based on electronic health records (EHR) [7, 8].

In this study, we compared patient-reported information on symptoms of anxiety, depression, and QoL domains, with data for similar constructs registered in the patients' EHR. We used data from a previous study that collected PROs data from a convenience sample of women with and without history of breast cancer [9], and for whom EHR data were available.

For peer review only

For peer review only - http://bmjopen.bmj.com/site/about/guidelines.xhtml

Methods

Study design and sampling frame

We used a convenience sample of women with PRO data available from a previous study [9]. For the original study, primary care practices contributing with data to the Clinical Practice Research Datalink (CPRD) GOLD primary care database in August 2018 were invited to participate. CPRD GOLD includes EHR of patients attending general practices in the UK that use Vision software to manage patient's records. Data are entered in the patients' EHR by GPs during consultations using Read codes [10], which include information on symptoms, diagnoses, and prescriptions [11]. The study protocol (Supplementary Materials) provides the sample size calculations for the original study. Patients registered with primary care practices that accepted to participate were considered potentially eligible for the study.

Patient eligibility criteria, selection and recruitment

A full description of eligibility and recruitment has been published elsewhere [9]. Briefly, inclusion criteria for the breast cancer survivors' group were 1) diagnosis of invasive breast cancer at least one year before (all stages) and 2) aged 18-80 years. To ensure that the recorded breast cancer was incident, we required one year of follow-up in CPRD prior to the breast cancer diagnosis. For the comparison group, inclusion criteria were 1) no history of cancer (except non-melanoma skin cancer), 2) aged 18-80 years, and 3) at least 2 years of follow-up data in CPRD (since we required one year of follow-up before and after cancer to be included in the breast cancer group). Exclusion criteria for both groups were 1) inability to complete a self-reported questionnaire (e.g. due to dementia) and 2) having had another (non-breast) cancer or having been treated for a non-invasive breast tumour.

The CPRD GOLD primary care database was used to identify all breast cancer survivors from participating practices, and a random sample of women with no prior cancer (frequency matched on age to breast cancer survivors) from the same practices. GPs applied inclusion and exclusion criteria (*vide* above), and sent the study materials to the eligible patients' addresses with a pre-paid envelope to return the questionnaires. Patients were recruited between January and November 2019.

Patient-reported outcomes

Anxiety and depression

For peer review only

Anxiety and depressive symptoms were measured with the Hospital Anxiety and Depression Scale (HADS) [12]. This is a 14-item self-reported screening tool for anxiety and depressive symptoms in the past week [12]. Based on their responses, we categorised patients as non-case (scores 0-7), borderline (scores 8-10) and probable case (scores 11-21) [12].

The QoL impact of anxiety and depression were measured with the respective domains in the Quality of Life in Adult Cancer Survivors Scale (QLACS) (see below).

Quality of life

QoL was assessed with QLACS [13]. This tool includes 47 items, divided in 7 generic domains (i.e. negative feelings; positive feelings; cognitive problems; pain; sexual function/interest; energy/fatigue; and social avoidance), and 5 cancer-specific domains (i.e. financial problems; benefits of cancer; distress-family; appearance; distress-recurrence) which are not considered further in this paper. Of the 7 generic QoL domains, 6 were considered suitable for comparison with data in the EHR because women with distress for these domains may visit their GP to seek help: 'negative feelings', 'cognitive problems', 'pain', 'sexual problems', 'fatigue' and 'social avoidance'. Each domain considered has 4 items on the QLACS questionnaire. Participants are instructed to answer in relation to the previous four weeks. Responses to each item are given on a Likert-type of scale that varies between 1 (never) and 7 (always); higher scores indicate poorer QoL.

To identify women who had high levels of distress for each domain, we calculated the mean response (i.e. the sum of the individual item scores divided by four; mean values range between one and seven). We considered a mean of ≥5 (corresponding to average replies of frequently, very often or always experiencing the stated symptom) to reflect distress in that domain. This was varied in sensitivity analyses (see below).

Outcomes recorded in electronic health records primary care data

We extracted the primary care EHR data for all participants. As PROs were collected between January and November 2019, we extracted data from the January 2020 version of CPRD GOLD, which included data from 1987 up to December 2019.

Anxiety and depression were defined using lists of Read codes from a systematic review [14]. For the QoL domains, we produced lists of Read codes closely related to the items in the QLACS domain (Supplementary Table 1). Read codes were used to identify women with these codes registered in their EHR in the 3, 6, 12 and 24 months prior to the date of last data collection from the practice. The last collection date varied from practice to practice, but was generally within three weeks of the database version (e.g. in the January 2020 version, the

For peer review only - http://bmjopen.bmj.com/site/about/guidelines.xhtml

date of last data collection from the practices was in median 20 days (inter-quartile range: 19-20) prior to 31 December 2019).

Data analysis

We calculated the proportion of women who reported high levels of distress in the questionnaires and had similar information in their EHR (i.e. sensitivity of the EHR for capturing patient-reported distress). To better understand the agreement between PROs and the EHR data, as a secondary analysis, we also calculated the proportion of women with codes indicating distress on each domain in their EHR that reported distress levels in the questionnaires (positive predictive value of the EHR for capturing patient-reported distress). Results were shown in tables and descriptively.

Sensitivity analysis

As we used an arbitrary cut-off to identify patients with poor QoL (mean domain-specific score of ≥5), two sensitivity analyses were conducted: 1) using a lower cut-off of ≥3; 2) considering a score of ≥5 on at least one item in the domain (rather than the mean) as reflecting distress. Finally, we explored whether breast cancer survivors had different results compared to women with no history of cancer.

Patient and public involvement

The authors are thankful to the cancer survivors involved with the Independent Cancer Patients' Voice (http://www.independentcancerpatientsvoice.org.uk/), a patient advocate group, for their comments on the study protocol.

For peer review only - http://bmjopen.bmj.com/site/about/guidelines.xhtml

Results

Characteristics of the participants

608 women from 40 primary care practices participated in the study (Table 1). General practices were from all four UK countries, but there was a predominance of practices from Scotland (N=16) and Wales (N=15) (Supplementary Table 2). The median number of consultations in 2018 and 2019 was 11, similar between breast cancer survivors (median 11, inter-quartile range (IQR): 7-16) and women with no history of cancer (median 11, inter-quartile range (IQR): 7-20). A quarter of the women had a higher education degree.

Table 1. Characteristics of the study participants*

	All participants (N=608)	
	N	%
**Age at completion of questionnaire**		
34-59 years	174	28.6
60-69 years	210	34.5
≥70 years	224	36.8
**Highest education level**		
Up to GCSEs, O levels, or equivalent	205	33.7
A levels or equivalent	65	10.7
Trade or technical training	106	17.4
Undergraduate or post-graduate degree	160	26.3
Did not want to disclose	72	11.8
**Ethnicity**		
White	589	96.9
Asian / Asian British	7	1.2
Did not want to disclose	12	2.0
**IMD quintile**		
1 (least deprived)	124	20.4
2	90	14.8
3	81	13.3
4	239	39.3
5 (most deprived)	74	12.2
**Living arrangements**		
Not alone	458	75.3
Alone	138	22.7
Did not want to disclose	12	2.0
**Country**		
England	114	18.8
Northern Ireland	49	8.1
Scotland	188	30.9
Wales	257	42.3

IMD = Index of Multiple Deprivation

* Proportion may not add to 100% due to rounding

For peer review only

Anxiety and depression

Of the 599 women that replied to the anxiety subscale, 242 (40%) had borderline to abnormal symptoms (Table 2). Borderline to abnormal symptoms of depression were also reported by 92 (15%) of the 601 women that replied to the subscale for depression. Almost no women had Read codes for anxiety or depression registered in their EHR in the 24 months prior. However, 108/242 (45%) of those reporting anxiety symptoms were prescribed with an anxiolytic or antidepressant (for anxiety), and 51/92 (55%) of those reporting depression symptoms were prescribed an antidepressant.

In the QoL scale, 100 of the 608 (17%) women that replied to questionnaire had average replies of frequently, very often or always experiencing negative feelings (mean score ≥5). Only 1 patient (1%) had Read codes related to anxiety and/or depression recorded in their EHR in the 24 months prior, but 51 (51%) had an antidepressant and/or anxiolytic prescription.

Of the patients that had information about negative feelings in their EHR, only a minority reported distress in the questionnaires (Supplementary table 3).

Other QoL domains: cognitive problems, fatigue, pain, sexual dysfunction, social avoidance

93/608 (16%) women reported high levels of distress related to cognitive problems, 156 (26%) to sexual dysfunction, 157 (26%) to fatigue/energy, and 82 (14%) to social avoidance (Table 3). No codes relevant to these domains were found in the patients' EHR up to 24 months prior. Using lower cut-offs to classify patients based in their QoL scores yielded similar results. Distress with pain was reported in the questionnaires by 123 (21%) of the women, and 65 (53%) of these had symptoms of pain and/or analgesic prescription recorded their EHR in the previous three months; this increased to 92 (75%) when a longer 24-month time window was used.

Sensitivity analyses

Results were similar to those of the main analysis when different criteria were used to define distress (see Table 2 and Table 3). There were no meaningful differences in the results between breast cancer survivors and women with no history of cancer (see Supplementary Table 4 and Supplementary Table 5).

For peer review only - http://bmjopen.bmj.com/site/about/guidelines.xhtml

Table 2. Capture of patient-reported anxiety and depression in patients' primary care records

Patient reported outcomes			Read codes for symptoms/diagnoses in the patients' electronic health records by time prior to LCD								Relevant drug prescription by time prior to LCD *							
			3mo		6mo		12mo		24mo		3mo		6mo		12mo		24mo	
	N	%	N	%	N	%	N	%	N	%	N	%	N	%	N	%	N	%
**HADS**																		
**Anxiety**																		
Normal	357	59.6	0	0	0	0	0	0	0	0	53	14.9	55	15.4	67	18.8	84	23.5
Borderline	124	19.7	0	0	0	0	0	0	0	0	33	28.0	34	33.9	40	33.9	49	41.5
Abnormal	118	20.7	1	0.8	1	0.8	2	1.6	2	1.6	40	32.3	42	28.8	53	42.7	59	50.0
**Depression**																		
Normal	509	84.7	0		0		0		0		75	14.7	77	15.1	96	18.9	120	23.6
Borderline	66	4.3	0		0		0		0		26	39.4	26	39.4	30	45.5	34	51.5
Abnormal	26	11.0	0		0		0		0		13	50.0	13	50.0	16	61.5	17	65.4
**QLACS**																		
**Negative feelings**																		
≥5	100	16.8	1	1.0	1	1.0	1	1.0	1	1.0	36	36.0	37	37.0	47	47.0	51	51.0
≥3	386	64.7	1	0.3	1	0.3	2	0.5	2	0.5	89	23.1	90	23.3	109	28.2	129	33.4
1 item ≥5	227	37.3	1	0.4	1	0.4	1	0.4	1	0.4	65	28.6	66	29.1	82	36.1	95	41.9

PRO = Patient reported outcomes; HADS = Hospital Anxiety and Depression Scale; QLACS = Quality of Life in Adults Cancer Survivors Scale. LCD = last collection date for the practice; mo = months. * Anxiolytics or antidepressants for anxiety; antidepressants for depression.

For peer review only

For peer review only - http://bmjopen.bmj.com/site/about/guidelines.xhtml

Table 3. Capture of patient-reported QoL-related distress in patients' primary care records

QoL Domain	Domain score	Patient reported outcomes No. §	%	Relevant Read codes in the electronic health record*, by time prior to the date of last data collection: 3mo No.	3mo %	6mo No.	6mo %	12mo No.	12mo %	24mo No.	24mo %
Cognitive problems	Average ≥5	93	15.5	0		0		0		0	
	Average ≥3	394	65.6	0		0		0		0	
	1 item ≥5	193	31.7	0		0		0		0	
Fatigue	Average ≥5	157	26.1	0		0		0		0	
	Average ≥3	472	78.5	0		0		0		0	
	1 item ≥5	536	88.2	0		0		0		0	
Physical pain	Average ≥5	123	20.6	65	52.8	70	56.9	82	66.7	92	74.8
	Average ≥3	330	55.4	106	32.1	116	35.2	152	46.1	186	56.4
	1 item ≥5	231	38.0	86	37.2	94	40.7	120	52.0	142	61.5
Sexual dysfunction	Average ≥5	156	25.7	0		0		0		0	
	Average ≥3	377	62.0	0		0		0		0	
	1 item ≥5	304	50.0	0		0		0		0	
Social avoidance	Average ≥5	82	13.5	0		0		0		0	
	Average ≥3	294	48.4	0		0		0		0	
	1 item ≥5	196	32.2	0		0		0		0	

QoL = quality of life; mo = months. * Severe cognitive dysfunction was an exclusion criterion for the study. § 608 women participated in the study; due to missing data for some items, the number of women included in the denominator varies slightly by domain. *Relevant Read codes were codes for cognitive impairment, dementia and dementia specific drugs (cognitive problems domain); low energy, tiredness (fatigue domain); pain, pain syndromes, analgesics prescriptions (pain domain); low libido, anorgasm, vaginismus (sexual dysfunction); social isolation and avoidance (social avoidance domain).

For peer review only - http://bmjopen.bmj.com/site/about/guidelines.xhtml

For peer review only

Discussion

Summary

Most patients who reported clinically relevant symptoms of anxiety and depression, and distress with cognitive problems, fatigue, physical pain, sexual dysfunction and social avoidance, did not have clinical codes for these conditions in their primary care EHR. This suggests that in some cases GPs may be unaware of problems adversely affecting their patients' QoL. Our results may also be partly explained by inconsistent coding, as evident from the number of women in receipt of medications for anxiety and depression, despite no diagnostic codes being present in the EHR. In these cases, GPs were evidently aware of the patient's condition but had not entered a diagnostic code into the EHR, which could lead to misleading information when routine coded data are used for audit and research.

Strengths and limitations

The ability to compare PROs with data available in the EHR represents a unique strength of this study. To our knowledge, no previous study has reported on this comparison. However, this study had limitations. We did not collect data on the date of questionnaire completion or questionnaire return to the research team, and therefore we could identify, precisely, the consultations that corresponded to when the PROs were evaluated. As questionnaires were returned over a 9-month period, this could have affected our assessment of outcomes particularly in 3 to 6 months prior. However, even analyses looking at coding in the previous 24 months showed substantial under-recording of mental-health and QoL-related distress in coded primary care data. Our approach to identify patients experiencing distress on specific QLACS domains used score thresholds that were not validated. However, sensitivity analysis using different cut-offs showed generally the same patterns. CPRD only captures drugs prescribed to patients, and widely used drugs for pain and fatigue are sold over-the-counter. We assumed that anxiolytics and antidepressants were taken for anxiety/depression, but we cannot rule out that some were for other indications such as pain or insomnia. Our definition of fatigue did not include prescriptions, as we did not have information on what drugs were prescribed with the aim of ameliorating this condition. The comparison for cognitive problems was limited by the need to exclude patients unable to reply to a self-reported questionnaire, and we cannot rule out that GPs may have been overly strict in applying this exclusion criterion, excluding mild cognitive impairment. Our results are based on a convenience sample of breast cancer survivors and non-cancer controls and may not be generalisable to the general population; however, results were similar in our sensitivity analysis comparing results between the two groups, which is probably because women with history of breast cancer were on average 8 years post diagnosis and most likely not under active treatment for cancer. Half

For peer review only

of the patients in our sample had history of breast cancer, which may have been associated with closer monitoring, and therefore we could have underestimated the extent of the missed coding of these problems. However, four sensitivity analyses comparing those with and without prior cancer showed no major differences between groups. We compared PROs with information coded in the EHR; while most of patient care is coded using records, GPs sometimes use other methods to keep records (e.g. free text entry) which are not available to us. Similarly, some codes in the EHR are unspecific (e.g. mood observations) and we could not assign a correspondence to domains of QoL. Ford et al. [15] explored the reasons for differences in coding for mental health conditions in primary care, and found that GPs may prefer free text and use codes for symptoms or general codes instead of definitive diagnoses. Therefore, it is possible that we underestimated, in some cases, the awareness of the GP about the patients' wellbeing.

Comparison with existing literature

Only one in 3 patients that reported distressing levels of negative feelings had similar information recorded in their EHR in the previous 3 months. This is consistent with patients often not seeking primary care for anxiety and depression [16]. Approximately one-half of the women that reported poor QoL related to pain had related information in the EHR in the previous 3 months. This may be partly explained by patients self-treating pain with widely used over-the-counter treatments such as paracetamol and ibuprofen. Conversely, the higher recording of pain compared to negative feelings could be related to patients more often seeking help for concerns perceived as being amenable to treatment. Patients with a prescription of antidepressants / anxiolytics and that reported normal levels of depressive / anxiety symptoms are not unexpected – these drugs are effective at improving symptoms of depression and anxiety, but have long treatment courses and patients are recommended to continue pharmacological treatment for months after symptoms disappear to prevent relapse [17, 18].

We did not find any records of cognitive dysfunction, social avoidance, sexual dysfunction or fatigue in the EHR of the participating women in the previous 24 months. An absence of entries for social avoidance is plausible; Read codes for social avoidance have seldom been used in the database. A lack of records for sexual dysfunction is in keeping with evidence that only a small proportion of people contact GPs for issues related to sexual function [19]. The lack of coded records for cognitive dysfunction and fatigue was more unexpected. It is possible that GPs systematically excluded people with mild cognitive dysfunction. For fatigue, a manual review of all entries in the EHR of patients that reported distressing levels of fatigue revealed

For peer review only - http://bmjopen.bmj.com/site/about/guidelines.xhtml

a pattern of multimorbidity, almost always with diagnoses where fatigue is implicit (e.g., heart failure, chronic obstructive pulmonary disease), but no explicit codes for fatigue.

Implications for research and/or practice

It is important to raise awareness that patients may not always actively report their distress. Even when GPs are aware of health issues, they are not always coded in the patient record, and thus EHRs have low sensitivity to detect patients experiencing poor QoL at a particular point in time. Studies investigating anxiety and depression should consider prescriptions as well as clinical codes, as many patients were prescribed anxiolytics and antidepressants without having a Read code for these conditions.

Similarly to other studies [20], in this study the collection of PRO data was not, unfortunately, followed by feedback of the results to the patients or to the patients' GPs. This was because the authorisation to conduct this study within the UK National Health Service was granted on the basis that there would separation between the researchers and the identity of patients and GPs, and we could only access anonymised data. Krageloh et al., 2015 [20] highlight in their review that most studies where there was a formal procedure to feedback PRO results to patients and health care providers reported better outcomes in this group compared to controls [20]. Future studies of PRO outcomes in the NHS should explore options to report back results without violating the data protection regulation in place.

Conclusion

We found substantial under-recording of mental-health and QoL-related distress in coded primary care data. In addition, there may be inconsistent coding of known conditions, meaning that studies of mental-health and QoL-related outcomes using EHR databases likely underestimate the absolute burden of these outcomes in the population. Further research is needed to understand whether or not GPs are aware of patient distress in cases where codes have not been recorded.

For peer review only - http://bmjopen.bmj.com/site/about/guidelines.xhtml

Contributors

HC, RW, and KB designed the study. HC, RW, and KB obtained ethical approvals. HD, HC, RW, and KB managed data collection. HC entered the data and performed analyses. All authors revised the manuscript for important intellectual content and approved the final version of the manuscript.

Funding

H.C. was supported by an MRC Case Studentship in Epidemiology, jointly funded by UK Medical Research Council (MRC; https://mrc.ukri.org/) and the Clinical Practice Research Datalink (https://www.cprd.com/) at the Medicines and Healthcare Products Regulatory Agency (grant number MR/M016234/1 to H.C.). K.B. is funded by a Wellcome Senior Research Fellowship (grant number 220283/Z/20/Z) and previously held a Sir Henry Dale Fellowship jointly funded by Wellcome Trust (https://wellcome.ac.uk/) and the Royal Society (https://royalsociety.org/) (grant number 107737/Z/15/Z). The funders had no role in study design, data collection and analysis, decision to publish, or preparation of the manuscript.

Competing interests

Dr Bhaskaran reports grants from Wellcome Trust, the Royal Society, Medical Research Council, and British Heart Foundation, outside the submitted work. Dr Williams and Ms Dempsey report that CPRD has financial relationships with its clients, including the London School of Hygiene and Tropical Medicine, in relation to providing access to research data and services outside the submitted work. HC has no conflict of interest to disclose.

Ethics approval and consent to participate

This study was approved by the East of England - Cambridge South Research Ethics Committee (Ref: 17/EE/0403), the London School of Hygiene and Tropical Medicine Interventions Research Ethics Committee (Ref: 14417) and the Health Research Authority and Health and Care Research Wales (IRAS Project ID: 224561). Implicit patient consent was obtained when the patient posted the completed questionnaires.

For peer review only

Data availability statement

This study is based in part on data from the Clinical Practice Research Datalink obtained under licence from the UK Medicines and Healthcare products Regulatory Agency. The terms of our licence to access the data preclude us from sharing individual patient data with third parties. The electronic health records raw data may be requested directly from CPRD following their usual procedures.

Open Access

This research was funded in part by the Wellcome Trust [grant numbers 107737/Z/15/Z and 220283/Z/20/Z]. For the purpose of open access, the authors have applied a CC BY public copyright licence to any Author Accepted Manuscript version arising from this submission.

For peer review only

References

1. *Quality of Life - The assessment, analysis and reporting of patient-reported outcomes*. third ed. 2016, UK: John Wiley & Sons, Ltd.
2. Institute for Health Metrics and Evaluation. *GBD Compare: Years Lived with Disability, United Kingdom, 2019*. [cited 2021; Available from: https://vizhub.healthdata.org/gbd-compare/.
3. Patel, V., et al., *The Lancet Commission on global mental health and sustainable development.* Lancet, 2018. **392**(10157): p. 1553-1598.
4. Campion, J., et al., *Public mental health: required actions to address implementation failure in the context of COVID-19.* Lancet Psychiatry, 2022. **9**(2): p. 169-182.
5. Alonso, J., et al., *Treatment gap for anxiety disorders is global: Results of the World Mental Health Surveys in 21 countries.* Depress Anxiety, 2018. **35**(3): p. 195-208.
6. National Institute for Health and Care Excellence (NICE), *Common mental health problems: identification and pathways to care. Clinical guideline 123*. 2011.
7. Gray, J., D. Orr, and A. Majeed, *Use of Read codes in diabetes management in a south London primary care group: implications for establishing disease registers.* BMJ, 2003. **326**(7399): p. 1130.
8. Shephard, E., S. Stapley, and W. Hamilton, *The use of electronic databases in primary care research.* Fam Pract, 2011. **28**(4): p. 352-4.
9. Carreira, H., et al., *Quality of life and mental health in breast cancer survivors compared with non-cancer controls: a study of patient-reported outcomes in the United Kingdom.* J Cancer Surviv, 2021. **15**(4): p. 564-575.
10. Chisholm, J., *The Read clinical classification.* BMJ, 1990. **300**(6732): p. 1092.
11. Herrett, E., et al., *Data Resource Profile: Clinical Practice Research Datalink (CPRD).* Int J Epidemiol, 2015. **44**(3): p. 827-36.
12. Zigmond, A.S. and R.P. Snaith, *The hospital anxiety and depression scale.* Acta Psychiatr Scand, 1983. **67**(6): p. 361-70.
13. Avis, N.E., et al., *Assessing quality of life in adult cancer survivors (QLACS).* Qual Life Res, 2005. **14**(4): p. 1007-23.
14. Carreira, H., et al., *Identification of mental health and quality of life outcomes in primary care databases in the UK: a systematic review.* BMJ Open, 2019. **9**(7): p. e029227.
15. Ford, E., et al., *"You don't immediately stick a label on them": a qualitative study of influences on general practitioners' recording of anxiety disorders.* BMJ Open, 2016. **6**(6): p. e010746.
16. Clement, S., et al., *What is the impact of mental health-related stigma on help-seeking? A systematic review of quantitative and qualitative studies.* Psychol Med, 2015. **45**(1): p. 11-27.
17. National Institute for Health and Care Excellence (NICE), *Depression in adults: treatment and management. Clinical Guideline 222*. 2022.
18. National Institute for Health and Care Excellence (NICE), *Generalised anxiety disorder and panic disorder in adults: management (CG113)*. 2011.
19. Hinchliff, S., et al., *Seeking help for sexual difficulties: findings from a study with older adults in four European countries.* Eur J Ageing, 2020. **17**(2): p. 185-195.
20. Krägeloh, C.U., et al., *Using Feedback From Patient-Reported Outcome Measures in Mental Health Services: A Scoping Study and Typology.* Psychiatric Services, 2015. **66**(3): p. 224-241.

For peer review only - http://bmjopen.bmj.com/site/about/guidelines.xhtml

Supplementary Table 1 Matching between QoL domain and information in the EHRs.

QoL domain	QLACS Items	Search in the EHR for Read codes* related to:
Negative feelings	19 Bothered by mood swings<br>7 Felt blue or depressed<br>9 Worried about little things<br>24 Felt anxious	Depression and/or anxiety (disorders and symptoms), antidepressants, or anxiolytic prescription
Cognitive problems	3 Bothered by having a short attention span<br>4 Had trouble remembering things<br>2 Difficulty doing things requiring concentration<br>23 Bothered by forgetting what started to do	Cognitive impairment; cognitive dysfunction symptoms; dementia*; dementia-specific drug*.
Physical pain	13 Bothered by pain preventing activities<br>17 Mood disrupted by pain or its treatment<br>27 Pain interfered with social activities<br>21 Had aches or pains	Pain; painful conditions; prescriptions of analgesics.
Sexual problems	16 Lacked interest in sex<br>26 Avoided sexual activity<br>12 Dissatisfied with sex life<br>10 Bothered by inability to function sexually	Low libido; anorgasmia; vaginismus.
Fatigue	11 Lacked energy to do things wanted to<br>14 Felt tired a lot<br>1 Had energy to do things wanted to do<br>5 Felt fatigued	Low energy; tiredness.
Social avoidance	18 Avoided social gatherings<br>20 Avoided friends<br>25 Reluctant to meet new people<br>15 Reluctant to start new relationships	Social isolation, or social avoidance.

* Definitions were based on a comprehensive systematic review of the studies that defined mental health and quality of life-related outcomes in primary care databases of electronic health records [11].

QLACS = Quality of Life in Adult Cancer Survivors Scale; EHR = electronic health records

For peer review only

Supplementary Table 2 Characteristics of the practices that participated in the study.

	In this study N (%)	In CPRD database overall N (%)
**Number of practices by country**		
All	40 (100%)	971 (100%)
England	6 (15%)	573 (59.0%)
North West	2 (5.0%)	87 (9.0%)
London	1 (2.5%)	105 (10.8%)
South East	3 (7.5%)	73 (7.5%)
Northern Ireland	3 (7.5%)	42 (4.3%)
Scotland	16 (40.0%)	229 (23.6%)
Wales	15 (37.5%)	127 (13.1%)
Size of the practice, number of patients in 2018		
]2,500	1 (2.5%)	17 (1.8%)
[2,500-5,000[	4 (10.0%)	90 (9.3%)
[5,000-7,500[	9 (22.5%)	124 (12.8%)
[7,500-10,000[	14 (35.0%)	147 (15.1%)
[10,000-15,000[	8 (20.0%)	221 (22.8%)
[15,000-20,000[	3 (7.5%)	86 (8.9%)
≥20,000	1 (2.5%)	76 (7.8%)

For peer review only

Supplementary Table 3 Patient reported outcomes of women with codes related to distress in their primary care record.

			Patients with info for the domain in EHR		Patients scoring as distressed, according to patient-reported data					
					≥5		≥3		At least one item 5	
**Domain**	**Read codes related to:**	**Time prior to LDC**	**No. §**	**%**	**No.**	**%**	**No.**	**%**	**No.**	**%**
Negative feelings	Depression and/or anxiety (disorders and symptoms), antidepressants, or anxiolytic prescription.	3 mo.	118	19.4	36	31.3	89	77.4	65	55.1
		6 mo.	120	19.7	37	31.6	90	76.9	66	55.0
		12 mo.	146	24.0	47	33.1	109	76.8	82	56.2
		24 mo.	170	28.5	51	30.0	129	75.9	95	54.3
Cognitive problems	Cognitive impairment; cognitive dysfunction symptoms; dementia; dementia-specific drug*	3 mo.	0		-		-		-	
		6 mo.	0		-		-		-	
		12 mo.	0		-		-		-	
		24 mo.	0		-		-		-	
Fatigue	Low energy; tiredness.	3 mo.	0		-		-		-	
		6 mo.	0		-		-		-	
		12 mo.	0		-		-		-	
		24 mo.	0		-		-		-	
Physical pain	Pain; painful conditions; prescriptions of analgesics.	3 mo.	138	37.2	65	48.2	106	78.5	86	62.3
		6 mo.	150	24.7	70	47.6	116	78.9	94	62.7
		12 mo.	203	34.1	82	40.4	152	74.9	120	58.0
		24 mo.	264	43.4	92	35.5	186	71.8	142	53.8
Sexual dysfunction	Low libido; anorgasm; vaginismus.	3 mo.	0		-		-		-	
		6 mo.	0		-		-		-	
		12 mo.	0		-		-		-	
		24 mo.	0		-		-		-	
Social avoidance	Social isolation; social avoidance.	3 mo.	0		-		-		-	
		6 mo.	0		-		-		-	
		12 mo.	0		-		-		-	
		24 mo.	0		-		-		-	

EHR = electronic health records; QoL = quality of life; mo = months. * Severe cognitive dysfunction was an exclusion criterion for the study. § 608 women participated in the study; due to missing data for some items, the number of women included in the denominator varies slightly by domain.

For peer review only

Supplementary Table 4 Patient-reported outcomes from women with history of breast cancer and non-cancer controls.

			PROs				Patients scoring above a given threshold in the PRO study that had domain-related information in EHRs, by time prior to the last data collection for the practice															
		Mean domain cut-off	Breast cancer survivors		Non-cancer controls		Breast cancer survivors								Non-cancer controls							
							3mo		6mo		12mo		24mo		3mo		6mo		12mo		24mo	
**QLACS Domain**	**Read codes for**		**No. §**	**%**	**No. §**	**%**	**No.**	**%**	**No.**	**%**	**No.**	**%**	**No.**	**%**	**No.**	**%**	**No.**	**%**	**No.**	**%**	**No.**	**%**
Negative feelings	Depression and/or anxiety (inc. symptoms), antidepressants, or anxiolytic prescription	≥5	62	17.4	38	15.1	23	37.1	23	37.1	29	46.8	32	51.6	13	34.2	14	36.8	18	47.4	19	50.0
		≥3	231	64.9	155	61.5	58	25.1	58	25.1	70	30.3	82	35.5	31	20.0	32	20.7	39	25.2	47	30.3
		1 item ≥5	140	39.3	87	34.5	42	30.0	42	30.0	53	37.9	61	43.6	23	26.4	24	27.6	29	33.3	34	39.1
Cognitive problems	Cognitive dysfunction; dementia; dementia-specific drug.*	≥5	61	17.1	32	12.7	0		0		0		0		0		0		0		0	
		≥3	238	66.9	156	61.9	0		0		0		0		0		0		0		0	
		1 item ≥5	123	34.6	70	27.8	0		0		0		0		0		0		0		0	
Physical pain	Pain; painful conditions; prescriptions of analgesics.	≥5	72	20.2	51	20.2	65	52.9	70	56.9	82	66.7	92	74.8	28	54.9	30	58.8	37	72.6	40	78.4
		≥3	194	54.5	136	54.0	106	32.1	116	35.2	152	46.1	186	56.4	42	30.9	45	33.1	65	47.8	77	56.6
		1 item ≥5	144	40.5	87	34.5	86	37.2	94	40.7	120	52.0	142	61.5	33	37.9	36	41.4	47	54.0	52	59.8
Sexual dysfunction	Low libido; anorgasm; vaginismus.	≥5	107	30.1	49	19.4	0		0		0		0		0		0		0		0	
		≥3	234	65.7	143	56.8	0		0		0		0		0		0		0		0	
		1 item ≥5	196	55.1	108	42.9	0		0		0		0		0		0		0		0	
Fatigue	Low energy; tiredness.	≥5	104	29.2	53	21.0	0		0		0		0		0		0		0		0	
		≥3	283	79.5	189	75.0	0		0		0		0		0		0		0		0	
		1 item ≥5	318	89.3	218	86.5	0		0		0		0		0		0		0		0	
Social avoidance	Social isolation; social avoidance.	≥5	50	14.0	32	12.7	0		0		0		0		0		0		0		0	
		≥3	171	48.0	123	48.8	0		0		0		0		0		0		0		0	
		1 item ≥5	125	35.1	71	28.2	0		0		0		0		0		0		0		0	

EHR = electronic health records; QoL = Health-Related Quality of Life; mo. = month. * Severe cognitive dysfunction was an exclusion criterion for the study. § 608 women participated in the study; due to missing data for some items, the number of women included in the denominator varies slightly by domain.

For peer review only

Supplementary Table 5 Patient-reported outcomes of patients with codes related to distress in their primary care record, by history of breast cancer.

			Patients with info for the domain in EHR				Patients scoring as distressed, according to patient-reported data													
							Breast cancer survivors						Non-cancer controls							
			Breast cancer survivors		Non-cancer controls		≥5		≥3		At least one item 5		≥5		≥3		At least one item 5			
**Domain**	**Read codes related to:**	**Time prior to LDC**	**No.** §	**%**	**No.** §	**%**	**No.**	**%**	**No.**	**%**	**No.**	**%**	**No.**	**%**	**No.**	**%**	**No.**	**%**		
Negative feelings	Depression and/or anxiety (disorders and symptoms), antidepressants, or anxiolytics.	3 mo.	75	21.7	40	15.9	23	30.7	58	77.3	42	54.6	13	32.5	31	77.5	23	56.1		
		6 mo.	76	22.0	41	16.3	23	30.3	58	76.3	42	53.9	14	34.2	32	78.1	24	57.1		
		12 mo.	92	26.6	50	19.9	29	31.5	70	76.1	53	55.8	18	36.0	39	78.0	29	33.3		
		24 mo.	109	31.5	61	24.3	32	29.4	82	75.2	61	54.0	19	31.2	47	77.1	34	54.8		
Cognitive problems	Cognitive impairment; dementia; specific drugs*	3 mo.	0				-		-		-		-		-		-			
		6 mo.	0				-		-		-		-		-		-			
		12 mo.	0				-		-		-		-		-		-			
		24 mo.	0				-		-		-		-		-		-			
Physical pain	Pain; painful conditions; prescriptions of analgesics.	3 mo.	89	25.0	49	19.4	37	41.6	64	71.9	53	59.6	28	57.1	42	85.7	33	67.4		
		6 mo.	98	27.5	52	20.6	40	40.8	71	72.5	58	59.2	30	57.7	45	86.5	36	69.2		
		12 mo.	127	35.7	80	31.8	45	35.4	87	68.5	73	50.7	37	46.3	65	81.3	47	58.8		
		24 mo.	162	45.5	102	40.5	52	32.1	109	67.3	90	55.6	40	39.2	77	75.5	52	51.0		
Sexual dysfunction	Low libido; anorgasm; vaginismus.	3 mo.	0				-		-		-		-		-		-			
		6 mo.	0				-		-		-		-		-		-			
		12 mo.	0				-		-		-		-		-		-			
		24 mo.	0				-		-		-		-		-		-			
Fatigue	Low energy; tiredness.	3 mo.	0				-		-		-		-		-		-			
		6 mo.	0				-		-		-		-		-		-			
		12 mo.	0				-		-		-		-		-		-			
		24 mo.	0				-		-		-		-		-		-			
Social avoidance	Social isolation; social avoidance.	3 mo.	0				-		-		-		-		-		-			
		6 mo.	0				-		-		-		-		-		-			
		12 mo.	0				-		-		-		-		-		-			
		24 mo.	0				-		-		-		-		-		-			

EHR = electronic health records; QoL = Health-Related Quality of Life; mo. = month. * Severe cognitive dysfunction was an exclusion criterion for the study. § 608 women participated in the study; due to missing data for some items, the number of women included in the denominator varies slightly by domain.

For peer review only

Supplementary protocol

LONDON SCHOOL of HYGIENE & TROPICAL MEDICINE

Mental health and quality of life of female breast cancer survivors compared to women who did not have cancer: study protocol

Research team

Helena Carreira, London School of Hygiene & Tropical Medicine

Rachael Williams, Clinical Practice Research Datalink

Susannah Stanway, The Royal Marsden NHS Foundation Trust

Liam Smeeth, London School of Hygiene & Tropical Medicine

Krishnan Bhaskaran, London School of Hygiene & Tropical Medicine

London, February 2020

For peer review only

Summary

We aim to assess the quality of life (QoL), and presence and severity of anxiety and depressive symptoms, in women who have had breast cancer diagnosed at ≥1 year, compared to women who did not have cancer.

The Clinical Practice Research Datalink (CPRD) primary care database will be used to select a random sample of breast cancer survivors (≥1 year), whose general practitioner (GP) agrees to participate in the study (see below), and who were registered with the practice for ≥1 year before and after the breast cancer diagnosis. Age-matched women who never had cancer will be randomly selected from the same practice. Staff at each practice will mail the study materials to the eligible women, who will complete the questionnaires and send those to the CPRD Intervention Studies Team for processing.

In addition, a secondary objective of this study is to assess whether PROs can be reasonably studied by using electronic health records (EHR), as these would involve fewer resources. For this, the EHR of the participating women will be collated from the CPRD primary care database and the results will be compared to those reported by the patients.

For peer review only

For peer review only - http://bmjopen.bmj.com/site/about/guidelines.xhtml

Table of Contents

Summary **7**
1 Background **9**
2 Aims and objectives **11**
3 Plan of investigation **12**
3.1 Study type 12
3.2 Study site 12
3.3 Study design 12
3.4 Study population 12
3.5 Comparison group 12
3.6 Recruitment of the participants 12
4 Data to be collected **15**
4.1 Health-related quality of life 15
4.2 Anxiety and depression 15
4.3 Clinical and socio-demographic data 15
4.4 Deprivation measures 15
5 Data/statistical analysis **17**
6 Plan for addressing missing data **23**
7 Sample size **24**
8 Feasibility counts **27**
9 Pilot study **28**
10 Limitations of the study design, data sources and analytical methods **29**
11 Patient or user group involvement **30**
12 Plans for disseminating and communicating study results **31**
13 Timetable **32**
14 References **33**
15 Appendices **37**
Appendix 1. List of read codes to identify breast cancer patients [50]. 38
Appendix 2. Items of the Quality of Life in Adult Cancer Survivors Scale (QLACS) grouped by domain 39
Appendix 3. Quality of Life in Adult Cancer Survivors Scale (QLACS) [36] 41
Appendix 4. Generic domains of HRQoL [36] 43
Appendix 5. Hospital Anxiety and Depression Scale [37] 44
Appendix 6. Clinical information 45
Appendix 7. Demographic information 46

For peer review only

For peer review only - http://bmjopen.bmj.com/site/about/guidelines.xhtml

1 Background

Breast cancer is the most common malignancy diagnosed in women in the United Kingdom (UK), excluding non-melanoma skin cancer [1]. The five-year age-standardised net survival for patients diagnosed with breast cancer in 2005-09 was 81% [2]. Breast cancer survivors are the largest group of cancer survivors in the UK [3, 4]: approximately 570,000 women were estimated to be living with or beyond breast cancer in 2010; this corresponds to 1,803 per 100,000 women [4]. The increasing trends in incidence and survival [1, 2] suggest that the number of breast cancer survivors will continue to increase in the next decades [4].

Even though women now live longer after the breast cancer diagnosis, the disease is perceived as life threatening and a major cause of emotional distress [5]. Common reactions to the diagnosis include anxiety, feelings of loneliness, fear of death, hopelessness, anger, suicidal thoughts and existential issues [6, 7]. In addition to the sorrow of the diagnosis, most women undergo a long and complex journey of aggressive treatments [8] with iatrogenic effects that are likely to have a long-term negative impact on their mental health and health-related quality of life (HRQoL) [9, 10]. For example, surgery for tumour removal and lymph node status assessment may cause lymphoedema [11] and/or persistent pain [12], in addition to a life-long scar, which may change women's body image [13]. Chemotherapy may result in cognitive impairments [14, 15] and/or cause amenorrhea in pre-menopausal women, bringing fertility concerns (for women who want children) and vasomotor symptoms such as hot flushes, night sweats, breast sensitivity and/or pain [16, 17]. In the long-term, women also have to re-adapt to social and intimate relationships (including with their spouse [18] and offspring [19-21]), and deal with the fear of cancer recurrence and death [22].

Patients often report the social, mental and cognitive functioning as important outcomes of their disease [23-26]. However, few studies [27, **Error! Hyperlink reference not valid.**] focused on the mental health and HRQoL of large samples of cancer survivors in the UK. The Clinical Practice Research Datalink (CPRD) primary care database gathers data for consultations occurring in a large number of general practices in the UK. This database currently includes data for more than 11.3 million patients, from over 600 general practices [29]. The cohort of cancer survivors in this database is one of the largest in the world with data prospectively and routinely collected at primary care level. As most mental disorders are also managed at primary care level [30, 31], the CPRD primary care database offers a unique opportunity to study long-term mental disorders in women who have had breast cancer. The information available for some domains of HRQoL may also represent an opportunity to study what are normally patient reported outcomes at a much lower cost but there has been no study evaluating the extent to which EHR data can be reasonably used to study HRQoL.

For peer review only - http://bmjopen.bmj.com/site/about/guidelines.xhtml

Khan *et al* used the CPRD primary care data to evaluate the pattern of consultations for anxiety and depression in 2003-2005, as well as the prescription of antidepressants and anxiolytics, among 16,938 breast cancer survivors (>5 years) and 67,649 women without breast cancer [27]. This study showed that breast cancer survivors had significantly increased odds of being prescribed antidepressants and anxiolytics but not of consulting for anxiety or depression, compared to women who did not have breast cancer [27]. The interpretation of these results is not straightforward because: 1) patients consulting for anxiety or depression are likely to represent the most severe cases, as these disorders, especially in the sub-threshold or milder severities, are often undiagnosed [31] and their burden underestimated; 2) cancer survivors may have more contact with the health services and be therefore more likely to be diagnosed and/or treated for anxiety or depressive symptoms, compared to women who did not have breast cancer; 3) antidepressants may also be prescribed to breast cancer survivors as treatment for hot flushes [32], one of the commonest side effects of endocrine treatments [33], and it is unclear if the frequency of prescription of antidepressants for hot flushes differs between women who have had breast cancer and women who never had cancer. Considering this, it is unclear how well the data registered in the EHR represent the burden of anxiety and depressive conditions in the population. In addition, a population-based cohort study conducted in Denmark described a significantly increased risk of depression in the first years after diagnosis, whose magnitude and significance reduced over time [34]. Corresponding estimates for the five years after the diagnosis are not available in the UK.

The aim of this study is to investigate the HRQoL, and the presence and severity of anxiety and depressive symptoms, in breast cancer survivors (>1 year) and in women who did not have cancer. A secondary objective of this study is to compare the outcomes reported by the patients to the data available in the EHR. In doing so, we will assess the feasibility of using EHR to study outcomes that are usually reported directly by patients.

For peer review only

For peer review only - http://bmjopen.bmj.com/site/about/guidelines.xhtml

2 Aims and objectives

Aims

The primary aim of this study is to investigate the health-related quality of life (HRQoL), and the presence and severity of anxiety and depressive symptoms, in female breast cancer survivors (>1 year) compared to women who did not have cancer.

The secondary aim is to assess the feasibility of studying outcomes that are usually reported directly be patients by relying on the EHR data.

Specific objectives

1. To describe cancer-specific measures of HRQoL in breast cancer survivors, and to explore the impact of demographic and clinical factors;
2. To compare measures of HRQoL between breast cancer survivors and women who did not have cancer and to evaluate the impact of clinical and demographic variables;
3. To compare the severity of anxiety and depressive symptoms in breast cancer survivors and in women who did not have cancer, and to assess the impact of demographic and clinical variables;
4. To compare patient reported HRQoL, and anxiety and depressive symptoms, with the information registered in the EHR for similar constructs.

For peer review only

For peer review only - http://bmjopen.bmj.com/site/about/guidelines.xhtml

3 Plan of investigation

3.1 Study type

Descriptive.

3.2 Study site

England, Wales, Scotland and Northern Ireland.

3.3 Study design

Cross-sectional.

3.4 Study population

Women aged 18 to 80 years old, diagnosed with a first primary cancer of the breast at one year or more ago at the recruitment date, and who had been registered for at least two years with a general practice contributing with 'up to standard' data to CPRD at the moment of the recruitment.

3.5 Comparison group

Adult women (18-80 years) without a previous cancer diagnosis, selected from the same primary care practices of the cancer patients.

3.6 Recruitment of the participants

Participants will be recruited from primary care practices contributing with data to the CPRD primary care database, via their GP. GPs working in practices considered 'active' (i.e. contributing with data to CPRD at the time of recruitment), and whose data quality at practice level has been judged as 'up to standard' by the CPRD internal quality procedures, will be invited by the CPRD Intervention Studies Team to participate in the study. Refusal to participate in the study will be recorded.

Breast cancer survivors

For peer review only - http://bmjopen.bmj.com/site/about/guidelines.xhtml

The EHR of the women registered with the GPs who accept to participate in the study will be collated. We will create a list of women who had a breast cancer recorded in the EHR using the list of Read codes provided in Appendix 1. We will then restrict the list to women aged 18-80 years, who were registered with the same primary care practice for at least one year before the breast cancer diagnosis, and who are currently alive, registered with the same practice, and have passed the first anniversary of their cancer diagnosis. A list of Read codes for other cancers [35] will be used to further exclude women who have had any other malignancy diagnosed before or after the breast cancer.

A random list of potentially eligible breast cancer survivors from each general practice will be selected. The number of women to be randomly selected from each practice will be calculated as the total number of women necessary for the study multiplied by the number of breast cancer survivors in the practice divided by the total number of potentially eligible breast cancer survivors in all practices.

The list of potentially eligible breast cancer survivors will be provided to the GP, and s/he will apply the following exclusion criteria:

a) The woman had a another cancer (not detected in the EHR), or has been treated for a non-invasive breast tumour;
b) The woman is considered unable to complete a self-administered questionnaire written in English for any reason.

The number of women excluded by the GP under each criterion will be recorded. Breast cancer survivors not excluded will be eligible for the study and invited to participate.

<u>Women who did not have cancer</u>

A list of Read codes [35] will be used to exclude patients who have had cancer from the list of patients attending the same practices as the cancer survivors. In addition, patients who have not been registered continuously for the last two years with the practice and outside the age range 18-80 years will be excluded. Women still in the list are potentially eligible.

The number of women to be selected from each practice will be calculated as: total number of women without cancer necessary for the study times the number of women without cancer in the practice divided by the total number of women without cancer in all practices.

For each practice, we will then calculate the proportion of breast cancer survivors in the following age groups: 18-24, 25-34, 35-44, 45-54, 55-64, 65-74, 75-80. The final list of

For peer review only - http://bmjopen.bmj.com/site/about/guidelines.xhtml

potentially eligible controls will be created by randomly selecting women with the same age distribution as of the breast cancer survivors of that same practice.

This list of potentially eligible controls will be sent to the GPs, and s/he will confirm that the women did not have a cancer and apply exclusion criteria a) and b).

Women not excluded will be considered eligible controls and invited to participate in the study.

For peer review only

For peer review only - http://bmjopen.bmj.com/site/about/guidelines.xhtml

4 Data to be collected

4.1 *Health-related quality of life*

Information on HRQoL will be collected using the Quality of Life in Adult Cancer Survivors Scale (QLACS) [36]. The QLACS was developed to take into account the specific needs of long-term cancer survivors, including issues that continue after treatment, new issues that arise during the period post-cancer, late physical effects of the cancer treatments and positive aspects of surviving to cancer [36]. It includes 47 items, divided in 7 generic and 5 cancer-specific domains (Appendix 2).

Breast cancer survivors will be asked to reply to all 47 items of the QLCAS (Appendix 3). Women who never had cancer will reply to the 28 items of the generic domains (Appendix 4).

4.2 *Anxiety and depression*

Data on anxiety and depressive symptoms will be collected with the Hospital Anxiety and Depression Scale (HADS, 5) [37]. This is a 14-item self-reported screening tool for anxiety and depressive symptoms in the past week. It contains two sub-scales, one for anxiety (HADS-A) and another for depression (HADS-D), with 7 items each [37]. This scale has been validated for use in primary care [38] and was used in primary care studies in the UK [39-41].

4.3 *Clinical and socio-demographic data*

Breast cancer survivors will be asked to provide information about the type of treatments received, the stage of their disease at diagnosis, the time since the last treatment (excluding long-term hormonal therapy), their menopausal status before and after the treatment, and how the cancer responded to the treatment (Appendix 6).

For all women, we will also collect data on potential confounders of the association between cancer history and mental health outcomes: education, ethnicity and social support (Appendix 7). Information on other potential confounders, such as co-morbidities or age at diagnosis will be obtained from the EHR.

4.4 *Deprivation measures*

The CPRD GOLD primary care data will be linked to the Index of Multiple Deprivation data. The IMD is an ecological measure based on the premise that deprivation can be measured

For peer review only - http://bmjopen.bmj.com/site/about/guidelines.xhtml

by different dimensions at small area level, and that individuals living in these areas share these dimensions of deprivation. The IMD is calculated for small geographical areas including approximately 1,500 residents, which are known as Lower-layer Super Output Areas (LSOA). Based on the 2011 Census, there were 32,844 LSOA in England. Mathematically, the IMD is calculated by using a set of indicators (at LSOA level) to produce information for seven domain indices that are related to material deprivation (income deprivation; employment deprivation; education, skills and training deprivation; health deprivation and disability; crime; barriers to housing and services; and living environment deprivation). The data from these seven domains are then combined using specified weights to produce a single measure of deprivation for each LSOA. The 32,844 LSOA are then sorted by measure of deprivation, and assigned a rank from 1 to 32,844, creating a relative measure of deprivation.

All GP practices contributing with data to the CPRD GOLD primary care database can be assigned IMD rank based on the GP practice post-code. This has been used in several studies as a proxy measure for socio-economic status at individual level because it is available for all patients, even though the ecological fallacy might apply (i.e. the individual experience may be different than the group). Patients can also be assigned an IMD rank based on their home address, but this is only available for the subset of patients that consent to the linkage scheme.

We will request practice postcode level of IMD for all GP practices participating in the study, and patient postcode level of IMD for all potentially eligible patients (in 20-quantiles, which may be later combined into narrower categories in analyses).

For peer review only

For peer review only - http://bmjopen.bmj.com/site/about/guidelines.xhtml

5 Data/statistical analysis

Proportion of participation and exclusions

The proportion of GPs and patients who accept to participate in the study will be calculated for the whole of the UK, by country within the UK, by region, and by quintiles of practice- and patient-postcode level of IMD.

The proportion of patients considered by the GP as ineligible will be reported separately for breast cancer survivors and women who did not have cancer.

The proportion of breast cancer survivors who accept to participate in the study will be calculated, as well as the proportion of women who did not have cancer. The denominator will include all women in each group to whom questionnaires were sent, even though we expect a minor proportion of envelopes returned because the patient may have moved or died, or the address may not be correct.

Objective 1: To describe cancer-specific measures of HRQoL in breast cancer survivors, and to explore the impact of demographic and clinical factors.

The QLACS includes 19 items for 5 cancer-specific domains of HRQoL (Appendix 2). Answers are provided on an ordinal Likert-type of scale, with values for individual items ranging from 1 to 7 [36]. For each breast cancer survivor, we will group the items by domain and calculate the sum of the individual scores under each domain [36]. All but one domain include 4 items; the "family distress" domain includes 3 items, and the sum of the individual scores will be rescaled to make the metric comparable with other domains. Values for each domain will range between 4 and 28. The range (minimum and maximum) scores will be reported for each domain, as well as the proportion of patients who score at the minimum and maximum values (floor and ceiling effects, respectively).

A mean or median score (depending on distribution) for each domain will be calculated from the individual-level sums of scores of the breast cancer survivors. Standard deviation will be calculated to quantify the dispersion of the data. The correlation coefficient among the mean scores of the domains will be reported.

For peer review only - http://bmjopen.bmj.com/site/about/guidelines.xhtml

A summary score for the cancer specific domains will be calculated by adding the mean/median scores of four domains (‘financial problems’, ‘distress-family’, ‘appearance’, and ‘distress-recurrence’); the mean/median score for ‘benefits from cancer’ is not included.

We will use linear regression models to estimate the association between the cancer-specific HRQoL domain scores and patients factors, such as stage at diagnosis or type of surgery. The dependent variable will be the sum of the individual items reported by each patient for that particular domain. The linear regression coefficients (β) from the regression models and the corresponding 95% confidence intervals will be reported.

Objective 2: To compare generic measures of HRQoL between breast cancer survivors and women who did not have cancer, and to evaluate the impact of demographic and clinical variables.

The QLACS includes 28 items for 7 generic domains of HRQoL, with values for individual items ranging from 1 to 7 [36] (Appendix 4).

The items will be grouped by domain (Appendix 2), and we will calculate, for each woman, the sum of the individual scores under each domain [36]. For each group of participants (i.e. breast cancer survivors and women who did not have cancer), the range (minimum and maximum) of the scores will be reported for each domain, as well as the proportion of women who score at the minimum and maximum values of the domain.

A mean or median score, depending on the distribution of the data, will be obtained for each group of women, by calculating the mean/median of the sum of the scores for each woman in that group. The respective standard deviation will be reported.

A summary score for the generic domains will be calculated as the sum of the individual domain scores.

The student’s two-sample t-test, or a non-parametric alternative if needed (i.e. Mann-Whitney distribution free test), will be used to assess the evidence for a difference in the summary scores for each domain between the two groups.

Linear regression will be used to evaluate the impact of cancer diagnosis on the mean scores of HRQoL, adjusting for potential confounders. The role of socio-economic and clinical variables will be explored. The model fit and the linear regression coefficients (β) will be reported as well as the 95% confidence intervals.

For peer review only - http://bmjopen.bmj.com/site/about/guidelines.xhtml

Objective 3: To compare the severity of depressive and anxiety symptoms in breast cancer survivors and in women who did not have cancer, and to assess the impact of clinical and demographic variables.

The Hospital Anxiety and Depression Scale contains two sub-scales, one for anxiety (HADS-A) and another for depression (HADS-D), with 7 items each [37]. Each item is rated from 0 to 3 and the total score for each sub-scale ranges between 0 and 21; higher scores represent higher symptoms of depression or anxiety [37].

To evaluate the severity of the depressive and anxiety symptoms in each group, the mean or median score, as appropriate, will be calculated for each sub-scale. The student's t-test or the Mann-Whitney test will be used to compare the mean/median score of depressive and of anxiety symptoms between cancer survivors and women who did not have cancer.

To identify patients with clinically relevant symptoms of depression or anxiety, the authors of the scale propose the cut-off of 0-7 for non-cases, 8-10 for borderline cases and 11-21 for probable cases, in both subscales.

The proportion of patients falling into the three categories (non-case, borderline, probable case) will be estimated for breast cancer survivors and for controls.

A chi-squared test will be used to assess whether there is evidence of differences in the proportion of patients in these categories between the two groups. A test for trend will be used to evaluate if there are increasing changes over the categories in each group.

The participants will then be categorised as having or not having clinically relevant levels of depressive or anxiety symptoms (cut off >10). Logistic regression models will be used to estimate the association between breast cancer history and clinically relevant levels of anxiety, and breast cancer history and clinically relevant symptoms of depression. The impact of clinical and demographic variables will be explored in the regression models. Crude and adjusted odds ratios, and respective 95% confidence intervals, will be reported.

Alexander et al. [42] evaluated the performance of the HADS as a screening test for major depressive disorder and anxiety in breast cancer survivors who were between 3 months and 2 years after main treatment conclusion (gold standard: non-patient Structured Clinical Interview for the Diagnostic and Statistical Manual of mental disorders (SCID)). Using the proposed cut-off of >10, the HADS-D had a sensitivity of 50% (95% confidence interval (95%CI): 27 to 73) and a specificity of 97% (95%CI: 93 to 99) [42]. However, the HADS-A had a sensitivity of 71% (95%CI: 30 to 95) and specificity of 87% (95%CI: 81 to 91) [42]. Even

For peer review only - http://bmjopen.bmj.com/site/about/guidelines.xhtml

though the optimal cut-off for this population has not been established, a sensitivity of 50% may be too low to be acceptable in clinical practice, and therefore we will conduct a sensitivity analysis considering the cut-off of ≥8 to classify women as having clinically relevant symptoms of anxiety or depression.

Objective 4: To compare the information reported by the patients for HRQoL, and for depressive and anxiety symptoms, with the information registered in the EHR for similar constructs

HRQoL

The QLACS includes seven generic domains of HRQoL (Appendix 4). Of these, five are particularly suitable for comparison with the data recorded in the EHR because women with distressing levels for these domains may have visited their GP to seek help: 'negative feelings', 'cognitive problems', 'physical pain', 'sexual problems' and 'fatigue'. Read codes for the 'social avoidance' domain are also available, and therefore we included also this domain.

For each woman, we will calculate the mean score for each domain (mean values will range between 1 and 7). Then, we will consider as reporting important levels of distress all women with a mean score of ≥5 (corresponding to replies of frequently, very often or always to most questions) in the domains of negative feelings, cognitive problems, physical pain, sexual problems and fatigue. Two sensitivity analyses will be conducted: 1) using a lower cut-off of ≥3 (corresponding to replies of sometimes and as often as not, in addition to replies of frequently, very often or always to most questions); 2) considering as exposed to important levels of distress all women who replied ≥5 to at least one item in the domain.

To identify evidence of the corresponding outcomes in the EHR, we will produce a list of Read codes closely related to the QLACS items for each domain (table 1). This list of Read codes will be used to identify women (who have had and who did not have breast cancer) with these outcomes registered in their EHR in the previous year (or since the first anniversary of diagnosis, if a cancer was diagnosed at less than 2 years).

For peer review only - http://bmjopen.bmj.com/site/about/guidelines.xhtml

Table 1 Domains and respective items of the QLACS scale, and conditions related to each domain.

Domain	Items in the QLACS	Read codes* related to:
Negative feelings	19 Bothered by mood swings;<br>7 Felt blue or depressed;<br>9 Worried about little things;<br>24 Felt anxious	Depression, anxiety
Cognitive problems	3 Bothered by having a short attention span<br>4 Had trouble remembering things<br>2 Difficulty doing things requiring concentration<br>23 Bothered by forgetting what started to do	Mild cognitive impairment<br>Cognitive dysfunction
Physical pain	13 Bothered by pain preventing activities<br>17 Mood disrupted by pain or its treatment<br>27 Pain interfered w/social activities<br>21 Had aches or pains	Pain reported as a symptom<br><br>Prescriptions of analgesics
Sexual problems	Sexual interest:<br>16 Lacked interest in sex<br>26 Avoided sexual activity<br><br>Sexual function<br>12 Dissatisfied w/sex life<br>10 Bothered by inability to function sexually	Sexual dysfunction<br>Hypoactive sexual disorder<br>Prescription of topical oestrogens
Fatigue	11 Lacked energy to do things wanted to<br>14 Felt tired a lot<br>1 Had energy to do things wanted to do<br>5 Felt fatigued	Fatigue<br>Low energy
Social avoidance	18 Avoided social gatherings<br>20 Avoided friends<br>25 Reluctant to meet new people<br>15 Reluctant to start new relationships	Social isolation<br>Social difficulties<br>Non aggressive unsocial conduct disorder

* This will also be based on the systematic review of the Read codes used to identify mental health outcomes in primary care databases.

For peer review only

We will estimate the proportion of women who reported distressing levels for these domains, and the proportion of women who have a recording of a similar construct in the EHR, separately for breast cancer survivors and for women who did not have cancer.

To estimate how much inquiring the patient adds to the information registered in the EHR, we will calculate the probability of:

1) having information for a particular domain registered in the EHR, among women who reported distressing levels for that domain (sensitivity);
2) not having any information registered in the EHR for a particular domain among women who did not report distressing levels for that domain (specificity);

For peer review only - http://bmjopen.bmj.com/site/about/guidelines.xhtml

3) reporting distressing levels for a particular domain among women who had information for that domain registered in the EHR (positive predictive value);
4) not reporting distressing levels for a particular domain among women who did not have data for that domain registered in the EHR (negative predictive value).

All probabilities will be calculated separately for breast cancer survivors and for women who did not have cancer.

Anxiety and depression

The scores of the HADS-A and HADS-D will be used to classify women as having clinically relevant levels of anxiety and of depressive symptoms, respectively, using >10 as cut-off. The proportion of women scoring above this threshold will be calculated.

Women with a diagnosis of an anxiety and/or depressive disorder will be identified in the EHR through a list of Read codes. This list will be based on a systematic review of the literature to identify mental disorders in primary care databases. Women with a Read code for a depressive or anxiety disorder diagnosed in the last year will be considered depressed or anxious. A sensitivity analysis will include Read codes for symptoms of depression and/or anxiety, to account for the difficulties in the diagnosis of these conditions.

We will calculate, for each group of women and for each disorder, the probability of:

1) having a diagnosis of anxiety/depression registered in the EHR among women who scored above the threshold in the HADS scale (sensitivity);
2) not having a diagnosis of anxiety/depression registered in the EHR among women who did not score above the threshold in the HADS scale (specificity);
3) scoring above the threshold in the HADS scale among women who had a diagnosis of anxiety/depression recorded in the EHR (positive predictive value);
4) not scoring above the threshold in the HADS scale among women who did not have a diagnosis of anxiety/depression recorded in the EHR (negative predictive value).

For peer review only

For peer review only - http://bmjopen.bmj.com/site/about/guidelines.xhtml

6 Plan for addressing missing data

We estimate that 5% of the women will have missing data for at least one item of the QLACS. This is a conservative estimate based on literature (the highest proportion of missing items was 3.2% [43]). The HADS has been shown to have excellent acceptability [37] and the proportion of missing items is usually small.

We will explore the pattern of missingness of the items by demographic and clinical variables. For that purpose, a variable will be created to denote records with incomplete information and we will explore the association between this variable and clinical and demographic variables. If the missingness can be explained by the other variables in the dataset, we will consider that it is missing at random, and specify a multiple imputation model to better represent the distribution from which the missing data came.

For peer review only

For peer review only - http://bmjopen.bmj.com/site/about/guidelines.xhtml

7 Sample size

We estimate that a sample of 260 breast cancer survivors and 260 women who did not have cancer are required to detect differences of the size reported in the literature. As participation rate in this type of studies has been low (approximately 20%), we believe that 1,400 women in each group need to be invited.

HRQoL

Table 2 provides details of the sample size calculation for the comparison of the summary scores of HRQoL, and of the mean scores of the generic domains of HRQoL, between breast cancer survivors and women who did not have cancer.

Table 2 Estimated sample size to compare the mean values of HRQoL in breast cancer survivors and women who did not have cancer.

	HRQoL mean score breast cancer survivors (SD)	HRQoL mean score normative data (SD)	Sample size per group †	Adjusted* sample size per group
Summary score	68.5 (22.7) [1]	60.9 (21.5)	133	800
Summary score	70.5 (26.6) [2]	60.9 (21.5)	100	600
Summary score	75.5 (26.3) [2]	60.9 (21.5)	43	350
**Generic domains**				
Negative feelings	9.7 (3.8)	7.1 (3.5)	31	300
Positive feelings	22.1 (4.7)	20.3 (6.3)	85	550
Cognitive problems	9.8 (5.0)	8.3 (2.7)	113	700
Sexual problems	11.8 (6.8)	9.0 (3.4)	58	400
Physical pain	9.7 (6.1)	7.8 (4.8)	131	800
Fatigue	11.8 (5.4)	10.3 (4.6)	**176**	**1,000**
Social avoidance	8.2 (4.3)	6.9 (2.8)	123	750

† Assuming an alpha of 0.05 and power of 80%.
* Calculated as the estimated sample size rounded upwards to the next 10 subjects (to take into account the uncertainty of the estimation process) divided by 0.2 (the estimated proportion of participation), and added a 100 patients to account for other variables to be studied.
[1] Women diagnosed with breast cancer at 18-24 months [44].
[2] Women diagnosed with breast cancer at 5 years of more [36, 43].

The summary mean scores for the generic domains of the QLACS among breast cancer survivors were obtained from the literature [36, 43, 44]. The mean/median scores of the generic domains among women who did not have cancer have not been reported. However, in a study involving long-term survivors of breast, bladder, head and neck, gynaecologic, prostate and colorectal cancer [36], patients with colorectal cancer ranked the lowest summary score (indicating better HRQoL) for the generic domains of HRQoL (mean 60.9, SD=21.5).

For peer review only - http://bmjopen.bmj.com/site/about/guidelines.xhtml

We used this score as a conservative estimate of the summary score of HRQoL in the general population, assuming that women who never have had cancer will not have worse HRQoL than the cancer patients who experience the best HRQoL. The same assumption was applied to estimate the sample size for the specific domains of HRQoL.

Anxiety and depression

Table 3 provides sample size estimates for the comparison of the mean scores of the two subscales of the HADS. As shown in the table, one study found a difference in mean HADS-Depression scores of just 0.6; to detect such a small difference would require 447 women per group, which would be beyond available resources. However, another study has calculated that differences of less than 1.4 in mean HADS-depression scores are not clinically important [45], and only around 75 patients per group would be required to detect differences above this level. For anxiety we would require 253 women per group to detect the minimum previously observed differences on the HADS scale.

Table 3 Estimated sample size to compare the mean scores of anxiety and depression between breast cancer survivors and women from the general population.

	Mean score breast cancer survivors (SD)	Mean score normative data (SD)	Sample size per group †	Adjusted* sample size per group
HADS-Anxiety	6.3 (2.8) [46]	4.8 (3.7) [46]	76	500
HADS-Anxiety	7.8 (3.0) [47]	7.1 (2.6) [47]	**253**	**1,400**
HADS-Depression	3.1 (3.3) [46]	3.7 (3.1) [46]	(447)	(2,350)
HADS-Depression	4.6 (3.3) [47]	3.2 (2.7) [47]	73	500

* Calculated as the estimated sample size rounded upwards to the next 10 subjects (to take into account the uncertainty of the estimation process) divided by 0.2 (the estimated proportion of participation), and added a 100 patients to account for other variables to be studied.

Table 4 provides estimates of the number of women necessary to compare the prevalences of anxiety and depression, as determined by the cut-offs of the HADS [48].

Table 4 Estimated sample size to compare the prevalences of anxiety and depression between breast cancer survivors and women from the general population.

Outcome	α	β	% of outcome in unexposed group [ref]	Estimated risk ratio [ref]	Sample size per group †	Adjusted* sample size per group
Anxiety	0.05	0.20	36.5 [48]	1.44 [48]	150	850
Depression	0.05	0.20	12.9 [48]	1.21 [48]	**2,614**	**13,170**

* Calculated as the estimate sample size rounded upwards to the next 10 subjects (to take into account the uncertainty of the estimation process) divided by 0.2 (the estimated proportion of participation), and added a 100 patients to account for other variables to be studied.

For peer review only - http://bmjopen.bmj.com/site/about/guidelines.xhtml

According to the calculations, over 13,000 breast cancer survivors and 13,000 women who did not have cancer would be needed to compare the prevalence of depression between the two groups of women. Recruiting more than 1,500 women for this study is not feasible, and therefore we chose the sample size necessary to compare the mean scores of anxiety and depression between the two groups (n=1,400 in each group, as outlined above and in Table 3).

For peer review only

For peer review only - http://bmjopen.bmj.com/site/about/guidelines.xhtml

8 Feasibility counts

A total of 43,704 women with breast cancer, and who were at least one year post-diagnosis, were identified in the July 2015 cut of the CPRD primary care database. Of these, 21,564 women had acceptable records from practices contributing with 'up to standard' data. A total of 8,763 women were still registered in practices that contributed with data to CPRD during the year of 2016, of which 7,498 (86%) were aged between 18 and 80 years old. Table 5 describes the distribution of the patients by region within England.

Table 5 Number of women and general practices with active records (as per June 2016) in the CPRD primary care database, by region.

Region code	Region label	No. of practices	No. of patients
1	North East	3	91
2	North West	28	1,139
3	Yorkshire & The Humber	3	160
4	East Midlands	0	0
5	West Midlands	18	763
6	East of England	10	557
7	South West	17	658
8	South Central	27	1,304
9	London	35	1,007
10	South East Coast	47	1,819
	Total	188	7,437

The estimated sample size (1,400) corresponds to 19% of the women potentially eligible for the study.

For peer review only

For peer review only - http://bmjopen.bmj.com/site/about/guidelines.xhtml

9 Pilot study

We will invite all GPs working in practices contributing with 'up to standard' data to CPRD at the time of recruitment to participate in the study.

Packages containing paper questionnaires will be sent to 140 breast cancer survivors and 140 women who did not have cancer (10% of those to be invited), randomly selected from the list of patients attending the first practices to sign up for the study. The pilot phase will run for 1 month. After that time, we will estimate:

1) the proportion of participation in each group;
2) the age distribution of the participants in each group;
3) the number of questionnaires with missing items.

Sample size calculations will be revised, if necessary. Afterwards, paper questionnaires will be sent out to the remainder of women to be invited, up to the estimated sample size.

For peer review only

For peer review only - http://bmjopen.bmj.com/site/about/guidelines.xhtml

10 Limitations of the study design, data sources and analytical methods

We will use the CPRD primary care database to classify women as exposed or not to breast cancer. CPRD has been shown to capture more than 90% of the cancer diagnoses registered in the cancer registries [49]. This is considered acceptable for this project, even though a small proportion of the women may be incorrectly classified as unexposed. We will request that the GP revises the list of patients to exclude potentially misclassified cases.

We expect a substantial proportion of patients to decline to participate in the study, as shown by the proportion of participation in previous studies. Selection bias may occur if the patients who accept to participate in the study differ systematically from those who do not. We will compare the demographic characteristics of the women who participate in the study with the broad characteristics of the women who had breast cancer in the CPRD primary care database. Also, we assumed a similar participation rate by age-group between women with breast with and without cancer. We will compare the age-distribution of the final samples and take age into account in multivariate analyses if necessary.

Women who are unable to complete a self-administered questionnaire due to advanced disease (e.g. terminally ill, patients with dementia or severe mental illnesses) will be excluded from the study. Therefore, the generalizability of our results will be limited women with a relatively good cognitive function.

The QLACS was validated in the United States but not in the UK population of cancer survivors. However, no translation is required and the entire scale will be applied, which makes unlikely the occurrence of substantial bias.

This study will have limited power to detect a strong association between having had a breast cancer and depression as defined by the cut-offs of the HADS scale. Our primary outcome will be the difference of the mean scores of each sub-scale, for which this study will have enough power.

For peer review only

For peer review only - http://bmjopen.bmj.com/site/about/guidelines.xhtml

11 Patient or user group involvement

Two women who never had cancer revised the invitation letter, participant information sheets and questionnaires for women in the non-cancer comparison group.

Breast cancer survivors identified through the Independent Cancer Patients' Voice (a patient advocate group and charity) revised the materials for breast cancer survivors. Comments from each group were incorporated into the study materials.

We will also ask selected members of the public and breast cancer survivors to comment on the report produced to share the study results prior to making these available.

For peer review only

For peer review only - http://bmjopen.bmj.com/site/about/guidelines.xhtml

12 Plans for disseminating and communicating study results, including the presence or absence of any restrictions on the extent and timing of publication

We plan to disseminate the results with the publication of an article in a peer-reviewed scientific journal. We will also present preliminary finding at scientific meetings.

To share the results with the general public, we will make the study results publicly available online. We will create a study's webpage on the website of the London School of Hygiene & Tropical Medicine. The website address for this webpage will be included in the participant information packs. A summary of findings from the study will be posted on the study webpage in due course. Anyone visiting this webpage (whether a participant, invitee, general practitioner or any interested member of the public) will be able to provide a contact email address through the webpage to subscribe for updates. The study researchers will use these contact email addresses for the sole purpose of letting interested parties know about updates to the study webpage.

For peer review only

For peer review only - http://bmjopen.bmj.com/site/about/guidelines.xhtml

13 Timetable

ACTIVITY	PLAN START	PLAN DURATION	ACTUAL START	ACTUAL DURATION	PERCENT COMPLETE	MONTH, 2018								MONTH, 2019						
						5	6	7	8	9	10	11	12	1	2	3	4	5	6	7
Ethical approvals	5	2			0%															
Recruitment of general practitioners	7	1			0%															
Identification of women potentially eligible for the study	7	2			0%															
Pilot phase for paper questionnaires (140 women)	8	1			0%															
Revison of sample size estimates and protocol	9	1			0%															
Data collection with paper questionnaires (1,400 women)	10	4			0%															
Data entry and cleaning	12	3			0%															
Data analysis	2	3			0%															
Drafting of newsletter for study participants	4	1			0%															
Drafting of scientific article for peer-reviewed journal	2	4			0%															

Plan Duration

End of data collection

only

For peer review only - http://bmjopen.bmj.com/site/about/guidelines.xhtml

14 References

1. Forman D, et al., eds. *Cancer Incidence in Five Continents, Vol. X*. IARC Scientific Publication No. 164. 2014, International Agency for Research on Cancer: Lyon.

2. Allemani, C., et al., *Global surveillance of cancer survival 1995-2009: analysis of individual data for 25,676,887 patients from 279 population-based registries in 67 countries (CONCORD-2).* Lancet, 2015. **385**(9972): p. 977-1010.

3. Ferlay, J., et al., *GLOBOCAN 2012 v1.0, Cancer Incidence and Mortality Worldwide: IARC CancerBase No. 11 [Internet].* 2013: Lyon, France: International Agency for Research on Cancer.

4. Maddams, J., M. Utley, and H. Moller, *Projections of cancer prevalence in the United Kingdom, 2010-2040.* Br J Cancer, 2012. **107**(7): p. 1195-202.

5. Weisman, A.D. and J.W. Worden, *The existential plight in cancer: significance of the first 100 days.* Int J Psychiatry Med, 1976. **7**(1): p. 1-15.

6. Schubart, J.R., et al., *Screening for psychological distress in surgical breast cancer patients.* Ann Surg Oncol, 2014. **21**(10): p. 3348-53.

7. Al-Azri, M., H. Al-Awisi, and M. Al-Moundhri, *Coping with a diagnosis of breast cancer-literature review and implications for developing countries.* Breast J, 2009. **15**(6): p. 615-22.

8. Senkus, E., et al., *Primary breast cancer: ESMO Clinical Practice Guidelines for diagnosis, treatment and follow-up.* Ann Oncol, 2015. **26 Suppl 5**: p. v8-30.

9. Caffo, O., et al., *Pain and quality of life after surgery for breast cancer.* Breast Cancer Res Treat, 2003. **80**(1): p. 39-48.

10. Bower, J.E., et al., *Fatigue in breast cancer survivors: occurrence, correlates, and impact on quality of life.* J Clin Oncol, 2000. **18**(4): p. 743-53.

11. Tsai, R.J., et al., *The risk of developing arm lymphedema among breast cancer survivors: a meta-analysis of treatment factors.* Ann Surg Oncol, 2009. **16**(7): p. 1959-72.

12. Andersen, K.G. and H. Kehlet, *Persistent pain after breast cancer treatment: a critical review of risk factors and strategies for prevention.* J Pain, 2011. **12**(7): p. 725-46.

13. Helms, R.L., E.L. O'Hea, and M. Corso, *Body image issues in women with breast cancer.* Psychol Health Med, 2008. **13**(3): p. 313-25.

14. Ahles, T.A., et al., *Neuropsychologic impact of standard-dose systemic chemotherapy in long-term survivors of breast cancer and lymphoma.* J Clin Oncol, 2002. **20**(2): p. 485-93.

15. Jim, H.S., et al., *Meta-analysis of cognitive functioning in breast cancer survivors previously treated with standard-dose chemotherapy.* J Clin Oncol, 2012. **30**(29): p. 3578-87.

16. Howard-Anderson, J., et al., *Quality of life, fertility concerns, and behavioral health outcomes in younger breast cancer survivors: a systematic review.* J Natl Cancer Inst, 2012. **104**(5): p. 386-405.

For peer review only

For peer review only - http://bmjopen.bmj.com/site/about/guidelines.xhtml

17. Thewes, B., et al., *Fertility- and menopause-related information needs of younger women with a diagnosis of early breast cancer.* J Clin Oncol, 2005. **23**(22): p. 5155-65.

18. Kim, Y., et al., *Quality of life of couples dealing with cancer: dyadic and individual adjustment among breast and prostate cancer survivors and their spousal caregivers.* Ann Behav Med, 2008. **35**(2): p. 230-8.

19. Cohen, M. and S. Pollack, *Mothers with breast cancer and their adult daughters: the relationship between mothers' reaction to breast cancer and their daughters' emotional and neuroimmune status.* Psychosom Med, 2005. **67**(1): p. 64-71.

20. Raveis, V.H. and S. Pretter, *Existential plight of adult daughters following their mother's breast cancer diagnosis.* Psychooncology, 2005. **14**(1): p. 49-60.

21. Hoving, J.L., M.L. Broekhuizen, and M.H. Frings-Dresen, *Return to work of breast cancer survivors: a systematic review of intervention studies.* BMC Cancer, 2009. **9**: p. 117.

22. Koch-Gallenkamp, L., et al., *Fear of Recurrence in Long-Term Cancer Survivors-Do Cancer Type, Sex, Time Since Diagnosis, and Social Support Matter?* Health Psychol, 2016.

23. Tiedtke, C., et al., *Survived but feeling vulnerable and insecure: a qualitative study of the mental preparation for RTW after breast cancer treatment.* BMC Public Health, 2012. **12**: p. 538.

24. Boykoff, N., M. Moieni, and S.K. Subramanian, *Confronting chemobrain: an in-depth look at survivors' reports of impact on work, social networks, and health care response.* J Cancer Surviv, 2009. **3**(4): p. 223-32.

25. Hunter, M.S., et al., *Menopausal symptoms following breast cancer treatment: a qualitative investigation of cognitive and behavioural responses.* Maturitas, 2009. **63**(4): p. 336-40.

26. Patrick D, Guyatt GH, and Acquadro C, *Chapter 17: Patient-reported outcomes. In: Higgins JPT, Green S (editors), Cochrane Handbook for Systematic Reviews of Interventions Version 5.1.0 (updated March 2011). The Cochrane Collaboration. Available from* http://www.cochrane-handbook.org. 2011.

27. Khan, N.F., et al., *Consulting and prescribing behaviour for anxiety and depression in long-term survivors of cancer in the UK.* Eur J Cancer, 2010. **46**(18): p. 3339-44.

28. Department of Health – Quality Health, *Quality of life of cancer survivors in England: Report on a pilot survey using Patient Reported Outcome Measures (PROMS)*. 2012.

29. Herrett, E., et al., *Data Resource Profile: Clinical Practice Research Datalink (CPRD).* Int J Epidemiol, 2015. **44**(3): p. 827-36.

30. National Institute for Health and Care Excellence (NICE), *Depression in adults with a chronic physical health problem: recognition and management (CG91)*. 2009.

31. National Institute for Health and Care Excellence (NICE), *Generalised anxiety disorder and panic disorder in adults: management (CG113)*. 2011.

For peer review only

For peer review only - http://bmjopen.bmj.com/site/about/guidelines.xhtml

32. Johns, C., et al., *Informing hot flash treatment decisions for breast cancer survivors: a systematic review of randomized trials comparing active interventions.* Breast Cancer Res Treat, 2016. **156**(3): p. 415-426.

33. Cella, D. and L.J. Fallowfield, *Recognition and management of treatment-related side effects for breast cancer patients receiving adjuvant endocrine therapy.* Breast Cancer Res Treat, 2008. **107**(2): p. 167-80.

34. Suppli, N.P., et al., *Increased risk for depression after breast cancer: a nationwide population-based cohort study of associated factors in Denmark, 1998-2011.* J Clin Oncol, 2014. **32**(34): p. 3831-9.

35. Bhaskaran, K., et al., *Body-mass index and risk of 22 specific cancers: a population-based cohort study of 5.24 million UK adults.* Lancet, 2014. **384**(9945): p. 755-65.

36. Avis, N.E., et al., *Assessing quality of life in adult cancer survivors (QLACS).* Qual Life Res, 2005. **14**(4): p. 1007-23.

37. Zigmond, A.S. and R.P. Snaith, *The hospital anxiety and depression scale.* Acta Psychiatr Scand, 1983. **67**(6): p. 361-70.

38. Wilkinson, M.J. and P. Barczak, *Psychiatric screening in general practice: comparison of the general health questionnaire and the hospital anxiety depression scale.* J R Coll Gen Pract, 1988. **38**(312): p. 311-3.

39. Thompson, C., et al., *Dimensional perspective on the recognition of depressive symptoms in primary care: The Hampshire Depression Project 3.* Br J Psychiatry, 2001. **179**: p. 317-23.

40. Ostler, K., et al., *Influence of socio-economic deprivation on the prevalence and outcome of depression in primary care: the Hampshire Depression Project.* Br J Psychiatry, 2001. **178**(1): p. 12-7.

41. Mallen, C.D. and G. Peat, *Screening older people with musculoskeletal pain for depressive symptoms in primary care.* Br J Gen Pract, 2008. **58**(555): p. 688-93.

42. Alexander, S., C. Palmer, and P.C. Stone, *Evaluation of screening instruments for depression and anxiety in breast cancer survivors.* Breast Cancer Res Treat, 2010. **122**(2): p. 573-8.

43. Avis, N.E., E. Ip, and K.L. Foley, *Evaluation of the Quality of Life in Adult Cancer Survivors (QLACS) scale for long-term cancer survivors in a sample of breast cancer survivors.* Health Qual Life Outcomes, 2006. **4**: p. 92.

44. Sohl, S.J., B. Levine, and N.E. Avis, *Evaluation of the Quality of Life in Adult Cancer Survivors (QLACS) scale for early post-treatment breast cancer survivors.* Qual Life Res, 2015. **24**(1): p. 205-12.

45. Puhan, M.A., et al., *The minimal important difference of the hospital anxiety and depression scale in patients with chronic obstructive pulmonary disease.* Health Qual Life Outcomes, 2008. **6**: p. 46.

46. Dahl, A.A., et al., *Arm/shoulder problems and insomnia symptoms in breast cancer survivors: cross-sectional, controlled and longitudinal observations.* Sleep Med, 2011. **12**(6): p. 584-90.

For peer review only - http://bmjopen.bmj.com/site/about/guidelines.xhtml

47. Calvio, L., et al., *Measures of cognitive function and work in occupationally active breast cancer survivors.* J Occup Environ Med, 2010. **52**(2): p. 219-27.

48. Boehmer, U., A. Ozonoff, and J. Potter, *Sexual Minority Women's Health Behaviors and Outcomes After Breast Cancer.* LGBT Health, 2015. **2**(3): p. 221-7.

49. Boggon, R., et al., *Cancer recording and mortality in the General Practice Research Database and linked cancer registries.* Pharmacoepidemiol Drug Saf, 2013. **22**(2): p. 168-75.

50. Ranopa, M., et al., *The identification of incident cancers in UK primary care databases: a systematic review.* Pharmacoepidemiol Drug Saf, 2015. **24**(1): p. 11-8.

For peer review only

For peer review only - http://bmjopen.bmj.com/site/about/guidelines.xhtml

15 Appendices

For peer review only

For peer review only - http://bmjopen.bmj.com/site/about/guidelines.xhtml

Appendix 1. List of read codes to identify breast cancer patients [50].

Read Code	Description
B34..11	CA FEMALE BREAST
B36..00	LOCAL RECURRENCE OF MALIGNANT TUMOUR OF BREAST
B340100	MALIGNANT NEOPLASM OF AREOLA OF FEMALE BREAST
B346.00	MALIGNANT NEOPLASM OF AXILLARY TAIL OF FEMALE BREAST
B341.00	MALIGNANT NEOPLASM OF CENTRAL PART OF FEMALE BREAST
B34y000	MALIGNANT NEOPLASM OF ECTOPIC SITE OF FEMALE BREAST
B34..00	MALIGNANT NEOPLASM OF FEMALE BREAST
B34z.00	MALIGNANT NEOPLASM OF FEMALE BREAST NOS
B343.00	MALIGNANT NEOPLASM OF LOWER-INNER QUADRANT OF FEMALE BREAST
B345.00	MALIGNANT NEOPLASM OF LOWER-OUTER QUADRANT OF FEMALE BREAST
B340.00	MALIGNANT NEOPLASM OF NIPPLE AND AREOLA OF FEMALE BREAST
B340000	MALIGNANT NEOPLASM OF NIPPLE OF FEMALE BREAST
B340z00	MALIGNANT NEOPLASM OF NIPPLE OR AREOLA OF FEMALE BREAST NOS
B34y.00	MALIGNANT NEOPLASM OF OTHER SITE OF FEMALE BREAST
B34yz00	MALIGNANT NEOPLASM OF OTHER SITE OF FEMALE BREAST NOS
B342.00	MALIGNANT NEOPLASM OF UPPER-INNER QUADRANT OF FEMALE BREAST
B344.00	MALIGNANT NEOPLASM OF UPPER-OUTER QUADRANT OF FEMALE BREAST
B347.00	MALIGNANT NEOPLASM, OVERLAPPING LESION OF BREAST
BB93.00	[M]COMEDOCARCINOMA NOS
BBM9.00	[M]CYSTOSARCOMA PHYLLODES, MALIGNANT
BB91100	[M]INFILTRATING DUCT AND LOBULAR CARCINOMA
BB91.00	[M]INFILTRATING DUCT CARCINOMA
BB9G.00	[M]INFILTRATING DUCTULAR CARCINOMA
BB9H.00	[M]INFLAMMATORY CARCINOMA
BB91000	[M]INTRADUCTAL PAPILLARY ADENOCARCINOMA WITH INVASION
BB94.00	[M]JUVENILE BREAST CARCINOMA
BB9F.00	[M]LOBULAR CARCINOMA NOS
BB9D.00	[M]MEDULLARY CARCINOMA WITH LYMPHOID STROMA
BB9K.00	[M]PAGET'S DISEASE AND INFILTRATING BREAST DUCT CARCINOMA
BB9K000	[M]PAGET'S DISEASE AND INTRADUCTAL CARCINOMA OF BREAST
BB9J.11	[M]PAGET'S DISEASE, BREAST
BB9J.00	[M]PAGET'S DISEASE, MAMMARY
BB94.11	[M]SECRETORY BREAST CARCINOMA
Byu6.00	[X]MALIGNANT NEOPLASM OF BREAST

For peer review only

For peer review only - http://bmjopen.bmj.com/site/about/guidelines.xhtml

Appendix 2. Items of the Quality of Life in Adult Cancer Survivors Scale (QLACS) grouped by domain

Domain	Item of the Quality of Life in Adult Cancer Survivors scale [36]
**Generic**	
Negative feelings	
	19 Bothered by mood swings
	7 Felt blue or depressed
	9 Worried about little things
	24 Felt anxious
Positive feelings	
	8 Enjoyed life
	28 Content with life
	6 Felt happy
	22 Had a positive outlook on life
Cognitive problems	
	3 Bothered by having a short attention span
	4 Had trouble remembering things
	2 Difficulty doing things requiring concentration
	23 Bothered by forgetting what started to do
Pain	
	13 Bothered by pain preventing activities
	17 Mood disrupted by pain or its treatment
	27 Pain interfered w/social activities
	21 Had aches or pains
Sexual interest	
	16 Lacked interest in sex
	26 Avoided sexual activity
Energy/fatigue	
	11 Lacked energy to do things wanted to
	14 Felt tired a lot
	1 Had energy to do things wanted to do
	5 Felt fatigued
Sexual function	
	12 Dissatisfied w/sex life
	10 Bothered by inability to function sexually
Social avoidance	
	18 Avoided social gatherings
	20 Avoided friends
	25 Reluctant to meet new people
	15 Reluctant to start new relationships

For peer review only

For peer review only - http://bmjopen.bmj.com/site/about/guidelines.xhtml

Domain	Item of the Quality of Life in Adult Cancer Survivors scale [36]
**Cancer-specific**	
Financial problems	
	43 Had money problems from cancer
	45 Financial problems from loss of income due to cancer
	30 Financial problems from cost of cancer surgery or tx
	37 Problems with insurance because of cancer
Benefits	
	40 Cancer helped recognize what important in life
	41 Better able to deal w/stress because of cancer
	32 Cancer helped cope better w/problems
	29 Appreciated life more because of cancer
Distress-family	
	34 Worried whether family had cancer causing genes
	31 Worried family members were at risk for cancer
	42 Worried family should have genetic tests - cancer
Appearance	
	35 Felt unattractive b/c of cancer or its treatment
	33 Self-conscious about appearance because of cancer
	44 Felt treated differently b/c of changes in appearance
	38 Bothered by hair loss from cancer treatments
Distress-recurrence	
	39 Worried about cancer coming back
	46 When felt pain, worried it was cancer again
	36 Worried about dying from cancer
	47 Preoccupied with concerns about cancer

For peer review only - http://bmjopen.bmj.com/site/about/guidelines.xhtml

Appendix 3. Quality of Life in Adult Cancer Survivors Scale (QLACS) [36]

We would like to ask you about some things that can affect the quality of people's lives. Some of these questions may sound similar, but please be sure to answer each one. Below is a scale ranging from never to always. Please indicate how often each of these statements has been true for you in the past four weeks. [Select one answer for each question]

		Never	Seldom	Sometimes	About as often as not	Frequently	Very often	Always
**In the past 4 weeks…**								
1	You had the energy to do the things you wanted to do.	1	2	3	4	5	6	7
2	You had difficulty doing activities that require concentrating.	1	2	3	4	5	6	7
3	You were bothered by having a short attention span.	1	2	3	4	5	6	7
4	You had trouble remembering things.	1	2	3	4	5	6	7
5	You felt fatigued.	1	2	3	4	5	6	7
6	You felt happy.	1	2	3	4	5	6	7
7	You felt blue or depressed.	1	2	3	4	5	6	7
8	You enjoyed life.	1	2	3	4	5	6	7
9	You worried about little things.	1	2	3	4	5	6	7
10	You were bothered by being unable to function sexually.	1	2	3	4	5	6	7
11	You didn't have energy to do the things you wanted to do.	1	2	3	4	5	6	7
12	You were dissatisfied with your sex life.	1	2	3	4	5	6	7
13	You were bothered by pain that kept you from doing the things you wanted to do.	1	2	3	4	5	6	7
14	You felt tired a lot.	1	2	3	4	5	6	7
15	You were reluctant to start new relationships.	1	2	3	4	5	6	7
16	You lacked interest in sex.	1	2	3	4	5	6	7
17	Your mood was disrupted by pain or its treatment.	1	2	3	4	5	6	7
18	You avoided social gatherings.	1	2	3	4	5	6	7
19	You were bothered by mood swings.	1	2	3	4	5	6	7
20	You avoided your friends.	1	2	3	4	5	6	7
21	You had aches or pains.	1	2	3	4	5	6	7
22	You had a positive outlook on life.	1	2	3	4	5	6	7
23	You were bothered by forgetting what you started to do.	1	2	3	4	5	6	7
24	You felt anxious.	1	2	3	4	5	6	7
25	You were reluctant to meet new people.	1	2	3	4	5	6	7
26	You avoided sexual activity.	1	2	3	4	5	6	7

For peer review only - http://bmjopen.bmj.com/site/about/guidelines.xhtml

		Never	Seldom	Sometimes	About as often as not	Frequently	Very often	Always
27	Pain or its treatment interfered with your social activities.	1	2	3	4	5	6	7
28	You were content with your life.	1	2	3	4	5	6	7
**The next set of questions asks specifically about the effects of your cancer or its treatment. Again, for each statement, indicate how often each of these statements has been true for you in the past four weeks.**								
29	You appreciated life more because of having had cancer.	1	2	3	4	5	6	7
30	You had financial problems because of the cost of cancer surgery or treatment.	1	2	3	4	5	6	7
31	You worried that your family members were at risk of getting cancer.	1	2	3	4	5	6	7
32	You realized that having had cancer helps you cope better with problems now.	1	2	3	4	5	6	7
33	You were self-conscious about the way you look because of your cancer or its treatment.	1	2	3	4	5	6	7
34	You worried about whether your family members might have cancer-causing genes.	1	2	3	4	5	6	7
35	You felt unattractive because of your cancer or its treatment.	1	2	3	4	5	6	7
36	You worried about dying from cancer.	1	2	3	4	5	6	7
37	You had problems with insurance because of cancer.	1	2	3	4	5	6	7
38	You were bothered by hair loss from cancer treatment.	1	2	3	4	5	6	7
39	You worried about cancer coming back.	1	2	3	4	5	6	7
40	You felt that cancer helped you to recognize what is important in life.	1	2	3	4	5	6	7
41	You felt better able to deal with stress because of having had cancer.	1	2	3	4	5	6	7
42	You worried about whether your family members should have genetic tests for cancer.	1	2	3	4	5	6	7
43	You had money problems that arose because you had cancer.	1	2	3	4	5	6	7
44	You felt people treated you differently because of changes to your appearance due to your cancer or its treatment.	1	2	3	4	5	6	7
45	You had financial problems due to a loss of income as a result of cancer.	1	2	3	4	5	6	7
46	Whenever you felt a pain, you worried that it might be cancer again.	1	2	3	4	5	6	7
47	You were preoccupied with concerns about cancer.	1	2	3	4	5	6	7

For peer review only - http://bmjopen.bmj.com/site/about/guidelines.xhtml

Appendix 4. Generic domains of HRQoL [36]

We would like to ask you about some things that can affect the quality of people's lives. Some of these questions may sound similar, but please be sure to answer each one. Below is a scale ranging from never to always. Please indicate how often each of these statements has been true for you in the past four weeks. [Select one answer for each question]

		Never	Seldom	Sometimes	About as often as not	Frequently	Very often	Always
**In the past 4 weeks…**								
1	You had the energy to do the things you wanted to do.	1	2	3	4	5	6	7
2	You had difficulty doing activities that require concentrating.	1	2	3	4	5	6	7
3	You were bothered by having a short attention span.	1	2	3	4	5	6	7
4	You had trouble remembering things.	1	2	3	4	5	6	7
5	You felt fatigued.	1	2	3	4	5	6	7
6	You felt happy.	1	2	3	4	5	6	7
7	You felt blue or depressed.	1	2	3	4	5	6	7
8	You enjoyed life.	1	2	3	4	5	6	7
9	You worried about little things.	1	2	3	4	5	6	7
10	You were bothered by being unable to function sexually.	1	2	3	4	5	6	7
11	You didn't have energy to do the things you wanted to do.	1	2	3	4	5	6	7
12	You were dissatisfied with your sex life.	1	2	3	4	5	6	7
13	You were bothered by pain that kept you from doing the things you wanted to do.	1	2	3	4	5	6	7
14	You felt tired a lot.	1	2	3	4	5	6	7
15	You were reluctant to start new relationships.	1	2	3	4	5	6	7
16	You lacked interest in sex.	1	2	3	4	5	6	7
17	Your mood was disrupted by pain or its treatment.	1	2	3	4	5	6	7
18	You avoided social gatherings.	1	2	3	4	5	6	7
19	You were bothered by mood swings.	1	2	3	4	5	6	7
20	You avoided your friends.	1	2	3	4	5	6	7
21	You had aches or pains.	1	2	3	4	5	6	7
22	You had a positive outlook on life.	1	2	3	4	5	6	7
23	You were bothered by forgetting what you started to do.	1	2	3	4	5	6	7
24	You felt anxious.	1	2	3	4	5	6	7
25	You were reluctant to meet new people.	1	2	3	4	5	6	7
26	You avoided sexual activity.	1	2	3	4	5	6	7
27	Pain or its treatment interfered with your social activities.	1	2	3	4	5	6	7
28	You were content with your life.	1	2	3	4	5	6	7

For peer review only - http://bmjopen.bmj.com/site/about/guidelines.xhtml

Appendix 5. Hospital Anxiety and Depression Scale [37]

[Omitted to preserve copyrights]

For peer review only

For peer review only - http://bmjopen.bmj.com/site/about/guidelines.xhtml

Appendix 6. Clinical information

1. What treatments have you received for your breast cancer? (Tick all that apply)

- ◻ Surgery
- ◻ Radiotherapy
- ◻ Chemotherapy (excluding hormone treatment)
- ◻ Hormone treatment
- ◻ Monoclonal antibodies / immunotherapy
- ◻ Don't know / can't remember

1.1 If you have had breast surgery, do any of the following apply to you? (Tick all that apply)

- ◻ I have had a lumpectomy (partial removal of the breast)
- ◻ I have had a mastectomy (complete removal of the breast)
- ◻ I have had a bilateral mastectomy (complete removal of the two breasts)
- ◻ I have had a breast reconstruction
- ◻ I am awaiting or considering breast reconstruction
- ◻ None of these apply to me
- ◻ Don't know / can't remember

2. At the time of the diagnosis, your cancer was:

- ◻ Localised to the breast only (without involving lymph nodes)
- ◻ Spread to the lymph nodes in the axilla
- ◻ Spread beyond the breast and the lymph nodes (metastatic)
- ◻ Don't know / can't remember

2.1 Please select your stage at diagnosis:

- ◻ Stage I
- ◻ Stage II
- ◻ Stage III
- ◻ Stage IV
- ◻ Don't know / can't remember

3. How long is it since you completed your initial cancer treatment?
(Treatment includes any chemotherapy, radiotherapy or surgery for your breast cancer. When answering this question please do not include hormone treatments such as Tamoxifen.)

- ◻ I am still having my initial treatment
- ◻ It is less than 3 months since my initial treatment
- ◻ It is between 3 and 12 months since my initial treatment
- ◻ It is between 1 and 5 years since my initial treatment
- ◻ It is more than 5 years since my initial treatment
- ◻ Don't know / can't remember

4. Regarding your menopausal status before and after the breast cancer diagnosis:

- ◻ My menstrual periods had finished when my cancer was diagnosed
- ◻ My menstrual periods finished during my treatments for breast cancer
- ◻ I had periods before the cancer diagnosis and continued to have them during/after the treatments
- ◻ Don't know / can't remember

5. Is your cancer currently in remission? (Complete remission means that there is no sign of cancer in your body)

- ◻ Yes
- ◻ No
- ◻ Don't know

For peer review only

For peer review only - http://bmjopen.bmj.com/site/about/guidelines.xhtml

Appendix 7. Demographic information

1. Which of these qualifications do you have?
- ◻ Up to GCSEs, O levels, or equivalent
- ◻ A levels or equivalent
- ◻ Undergraduate degree (for example BA, BSc)
- ◻ Post-graduate degree
- ◻ Trade, technical or vocational training
- ◻ Do not wish to disclose

2. What is your ethnic group?
- ◻ White
- ◻ Mixed / Multiple ethnic groups
- ◻ Asian / Asian British
- ◻ Black / African / Caribbean / Black British
- ◻ Other ethnic group
- ◻ Do not wish to disclose

3. Which statement best describes your living arrangements?
- ◻ I live with partner / spouse
- ◻ I live with family / friends
- ◻ I live alone
- ◻ I live in a nursing home or other long term care home
- ◻ Other
- ◻ Do not wish to disclose

For peer review only

For peer review only - http://bmjopen.bmj.com/site/about/guidelines.xhtml

STROBE Statement—Checklist of items that should be included in reports of ***cross-sectional studies***

	Item No	Recommendation	Location of information in the manuscript
**Title and abstract**	1	(*a*) Indicate the study's design with a commonly used term in the title or the abstract	Done, see title.
		(*b*) Provide in the abstract an informative and balanced summary of what was done and what was found	Done, see abstract.
**Introduction**			
Background/rationale	2	Explain the scientific background and rationale for the investigation being reported	p. 5
Objectives	3	State specific objectives, including any prespecified hypotheses	p. 5, lines 20-23
**Methods**			
Study design	4	Present key elements of study design early in the paper	p. 6, lines 2-10
Setting	5	Describe the setting, locations, and relevant dates, including periods of recruitment, exposure, follow-up, and data collection	p. 6, lines 2-28
Participants	6	(*a*) Give the eligibility criteria, and the sources and methods of selection of participants	p. 6, lines 12-22
Variables	7	Clearly define all outcomes, exposures, predictors, potential confounders, and effect modifiers. Give diagnostic criteria, if applicable	p. 7
Data sources/ measurement	8*	For each variable of interest, give sources of data and details of methods of assessment (measurement). Describe comparability of assessment methods if there is more than one group	pp. 6-8
Bias	9	Describe any efforts to address potential sources of bias	p. 7
Study size	10	Explain how the study size was arrived at	p. 6
Quantitative variables	11	Explain how quantitative variables were handled in the analyses. If applicable, describe which groupings were chosen and why	p. 8, lines 3-16
Statistical methods	12	(*a*) Describe all statistical methods, including those used to control for confounding	p. 8, lines 3-16
		(*b*) Describe any methods used to examine subgroups and interactions	p. 8, lines 3-16
		(*c*) Explain how missing data were addressed	p. 8
		(*d*) If applicable, describe analytical methods taking account of sampling strategy	n/a
		(*e*) Describe any sensitivity analyses	p. 8, lines 18-21
**Results**			
Participants	13*	(a) Report numbers of individuals at each stage of study—eg numbers potentially eligible, examined for eligibility, confirmed eligible, included in the study, completing follow-up, and analysed	p. 6, line 12
		(b) Give reasons for non-participation at each stage	p. 6, line 12
		(c) Consider use of a flow diagram	n/a

For peer review only - http://bmjopen.bmj.com/site/about/guidelines.xhtml
For peer review only

Descriptive data	14*	(a) Give characteristics of study participants (eg demographic, clinical, social) and information on exposures and potential confounders	Table 1.
		(b) Indicate number of participants with missing data for each variable of interest	Table 1.
Outcome data	15*	Report numbers of outcome events or summary measures	Tables 2 and 3.
Main results	16	(*a*) Give unadjusted estimates and, if applicable, confounder-adjusted estimates and their precision (eg, 95% confidence interval). Make clear which confounders were adjusted for and why they were included	n/a
		(*b*) Report category boundaries when continuous variables were categorized	n/a
		(*c*) If relevant, consider translating estimates of relative risk into absolute risk for a meaningful time period	n/a
Other analyses	17	Report other analyses done—eg analyses of subgroups and interactions, and sensitivity analyses	p. 10, lines 24-28
**Discussion**			
Key results	18	Summarise key results with reference to study objectives	p. 13, lines 2-11
Limitations	19	Discuss limitations of the study, taking into account sources of potential bias or imprecision. Discuss both direction and magnitude of any potential bias	p. 13, lines 12-35
Interpretation	20	Give a cautious overall interpretation of results considering objectives, limitations, multiplicity of analyses, results from similar studies, and other relevant evidence	p. 14, lines 1-19
Generalisability	21	Discuss the generalisability (external validity) of the study results	p.13, lines 27-29
**Other information**			
Funding	22	Give the source of funding and the role of the funders for the present study and, if applicable, for the original study on which the present article is based	p. 15

*Give information separately for exposed and unexposed groups.

Note: An Explanation and Elaboration article discusses each checklist item and gives methodological background and published examples of transparent reporting. The STROBE checklist is best used in conjunction with this article (freely available on the Web sites of PLoS Medicine at http://www.plosmedicine.org/, Annals of Internal Medicine at http://www.annals.org/, and Epidemiology at http://www.epidem.com/). Information on the STROBE Initiative is available at www.strobe-statement.org.

For peer review only
